# Optimality of Maximal-Effort Vaccination

**DOI:** 10.1007/s11538-023-01179-8

**Published:** 2023-06-23

**Authors:** Matthew J. Penn, Christl A. Donnelly

**Affiliations:** 1 Department of Statistics, University of Oxford, St Giles’, Oxford, OX1 3LB UK; 2 Department of Infectious Disease Epidemiology, Imperial College London, St Mary’s Campus, London, W2 1PG UK; 3 Pandemic Sciences Institute, University of Oxford, Roosevelt Drive, Oxford, OX3 7DQ UK

**Keywords:** Vaccination, Epidemiology, Epidemics, SIR Modelling

## Abstract

It is widely acknowledged that vaccinating at maximal effort in the face of an ongoing epidemic is the best strategy to minimise infections and deaths from the disease. Despite this, no one has proved that this is guaranteed to be true if the disease follows multi-group SIR (Susceptible–Infected–Recovered) dynamics. This paper provides a novel proof of this principle for the existing SIR framework, showing that the total number of deaths or infections from an epidemic is decreasing in vaccination effort. Furthermore, it presents a novel model for vaccination which assumes that vaccines assigned to a subgroup are distributed randomly to the unvaccinated population of that subgroup. It suggests, using COVID-19 data, that this more accurately captures vaccination dynamics than the model commonly found in the literature. However, as the novel model provides a strictly larger set of possible vaccination policies, the results presented in this paper hold for both models.

## Introduction

The COVID-19 pandemic has illustrated the importance of quickly implementing vaccination policies which target particular groups within a population (Fitzpatrick and Galvani 2021). The difference in final infections between targeted policies and uniform distribution to the entire population can be significant (Castro and Singer 2021; Estadilla et al. 2021) and so it is important that the models underlying these decisions provide realistic predictions of the outcomes of different policies.

One of the most commonly used models to forecast epidemics is the multi-group SIR (Susceptible–Infected–Recovered) model (Acemoglu et al. 2021; Kuniya 2019; Ram and Schaposnik 2021). This model divides the population into different groups based on characteristics such as age or occupation. Each group is then further sub-divided into categories of susceptible, infected and recovered. Where vaccination does not give perfect immunity, further sub-categorization based on vaccination status can also be used (Kuga and Tanimoto 2018), as will be done in this paper.

While many other approaches have been developed either by adding compartments to the SIR framework (Moore et al. 2021) or using completely different models such as networks (Chen and Sun 2014) or stochastic simulations (Ball and Lyne 2002), the multi-group SIR model remains popular because of its comparatively small number of parameters and its relatively simple construction and solution. In this paper, attention will thus be restricted to the multi-group SIR model, although it would be beneficial for future work to consider a wider range of disease models.

There are two general frameworks that are used to model optimal vaccination policies in a resource-limited setting. The first, used in papers such as Hill and Longini Jr (2003) and Becker and Starczak (1997), seeks to reduce the reproduction number, $R_0$ of the epidemic as much as possible by vaccinating before infections arrive in a population. It is simple to show that in this case, one should use all of the vaccinations available, and so this problem will not be considered further in this paper.

The second framework, used in papers such as Acemoglu et al. (2021) and Hansen and Day (2011) aims to minimise the total cost of an epidemic. This is the framework that will be discussed in this paper. The “cost” of an epidemic is, in general, defined to be the number of deaths (or equivalently, infections), with many papers also considering the cost of vaccination alongside the cost of other control measures, such as isolation, lockdown or treatment (Fu et al. 2022).

One important principle which underlies all of these vaccination policies is the acceptance that giving people their first dose of vaccine as soon as possible reduces the number of infections. Of course, this only holds when the timescale considered is sufficiently short for effects such as waning immunity and disease seasonality to be negligible, and a more complicated framework would be needed to model these effects. However, the acceptance of at least short-term optimality of maximal vaccination effort has been highlighted in the COVID-19 pandemic response, as countries began their vaccination programs as soon as vaccines became available (Mathieu et al. 2021).

To the best of the authors’ knowledge, no one has provided a mathematical proof that in a general, multi-group SIR model with imperfect vaccination, it is always best to vaccinate people as early as possible. Of course, it is not difficult to create a conceptually sound justification—vaccinating more people means that fewer people will catch the disease which will reduce the total number of infections. However, the SIR model is an approximation of the process of a disease spreading, and so it is important that it obeys this principle for all physical parameter values and vaccination policies.

Some special cases of the theorem presented in this paper have been previously proved in the literature. In particular, a significant number of papers have considered the optimal vaccination policy for a homogeneous population, with Abakuks (1972) first proving that, in this case, it is optimal to vaccinate at maximal effort (if one ignores the cost of vaccination). This proof held for vaccination policies that were finite sums of point mass “impulse” vaccinations, and has been generalised by papers such as Hansen and Day (2011), Zaman et al. (2008), Morton and Wickwire (1974) and Zhou et al. (2014) to a much wider class of vaccination policies, although the proof was still restricted to a single group and to perfect vaccination. Moreover, Hansen and Day (2011) notes that the case of imperfect vaccination (where vaccinated individuals can still get infected, although at a lower rate) remained a topic of open investigation, and so it can not easily be solved using the same methods presented in these papers. A slight extension is made in Duijzer et al. (2018) where it is shown that maximal effort is optimal in the case of perfect vaccination of any number of disconnected groups, but the full problem is still far from understood.

The general method of proof in the literature relies on Pontryagin’s Maximum Principle, which is difficult to apply to multi-group models due to the more complex structure of the equations. It is simple to characterise the solution in terms of the adjoint variables, as is done in Zhang et al. (2020) and Zavrakli et al. (2021) for a two-group model with imperfect vaccination, in Boutayeb et al. (2021) for a general *n*-group model with perfect vaccination and in Lee et al. (2012) for a six-group model with imperfect vaccination. However, determining whether this solution corresponds to the maximal effort solution in the case of zero vaccination cost requires the analysis of the adjoint ODE system, which is often just as complicated as the original disease model. In particular, the fact that vaccinated people need to be no more infectious, no more susceptible and be infected for no more time than unvaccinated people means that any analysis of the adjoint system would be complicated, as the properties of all the constituent parameters would need to be used.

Thus, in this paper, a novel approach is developed. Rather than attempting to use the general optimal control theory methodology, the specific structure of the SIR equation system is exploited. Using this, an inequality is derived which shows that if a given vaccination policy, $\tilde{{\varvec{U}}}$ vaccinates each individual at least as early as another vaccination policy, ${\varvec{U}}$, then the latter policy will lead to at least as many deaths (or equivalently, infections) as the former. As well as providing a constraint on the optimal solution, this theorem also highlights important structural properties of the model, as it shows that the number of deaths is everywhere non-increasing in the vaccination rates, rather than this just holding near the optimal solutions.

Also introduced in this paper is a more general model of vaccination than the one normally used in the literature. The one that is typically used (in almost all papers cited in this work such as Hansen and Day (2011), Zaman et al. (2008) and Kar and Batabyal (2011)) models decreasing vaccination uptake by assuming that the total number rate of people being vaccinated is the product of a vaccination rate and the proportion of susceptible people in the population. The model introduced in this paper allows for more flexibility in modelling the demand. However, the standard vaccination model is a special case of the general model introduced here, and so the theoretical results proved in this paper can be used by those following the standard model.

Alongside proving that the final infected, recovered and dead populations are non-increasing with increased and earlier vaccination effort, some cautionary contradictions to perhaps intuitive conjectures are also provided which show the importance of mathematical proof instead of simply intuition. In particular, it is shown that increased vaccination (under this model) can lead to, at a fixed finite time of the simulation, higher infection rates or a higher death count, despite the longer-term better performance of this policy. Indeed, it is results similar to these which make the proof of the optimality of maximal effort difficult, as it means that one must be very careful when constructing the inequalities that do hold for all models.

## Modelling

### Disease Transmission and Vaccination Model

Suppose that the population is divided into *n* subgroups, such that population of people in group *i* is $N_i$ and define

$$\begin{aligned} N:= \sum _{i=1}^n N_i. \end{aligned}$$

Define the compartments of people as follows, for $i = 1,...,n$:

$$\begin{aligned} S_i&:= \text {Number of people that are in group }i\text {, are susceptible, and are unvaccinated},\\ I_i&:= \text {Number of people that are in group }i\text {, are currently infected, and}\\&\qquad \quad \text {were infected while unvaccinated },\\ R_i&:= \text {Number of people that are in group }i\text {, are recovered or dead, and}\\&\quad \qquad \text {were infected while unvaccinated}, \\ S^V_i&:= \text {Number of people that are in group }i\text {, are susceptible and are vaccinated}, \\ I^V_i&:= \text {Number of people that are in group }i\text {, are infected}\\&\quad \qquad \text {and were infected after being vaccinated},\\ R^V_i&:= \text {Number of people that are in group }i\text {, are recovered or dead} \\&\quad \qquad \text {and were infected after being vaccinated}. \end{aligned}$$

This paper introduces a more general and flexible framework for vaccination, which is motivated as follows. It is assumed that there is a record of people who have received a vaccination and that protection from vaccination does not decay over time, so that no one is vaccinated more than once. Thus, if a total number, $U_i(t)dt$, of people in group *i* are given vaccines in a small time interval $(t,t+dt)$, and these vaccines are distributed randomly to the unvaccinated population in group *i*, the total population of susceptibles given vaccines in group *i* is

$$\begin{aligned} U_i(t)dt \times \text {P}\bigg (\text {A person in group }i\text { is in }S_i \vert \text { A person is in group }i\text { is unvaccinated}\bigg ) \end{aligned}$$

which is equal to

$$\begin{aligned} \frac{U_i(t)dt S_i(t)}{N_i - \int _0^tU_i(s)ds}, \end{aligned}$$

as $\int _0^tU_i(s)ds$ is the total population that are in group *i* and have been vaccinated before time *t*. For the remainder of this section, this vaccination model will be referred to as the “general” model

This results in the following model, based on SIR principles

1 $$\begin{aligned} \frac{dS_i}{dt}&= -\sum _{j=1}^n(\beta ^1_{ij}I_j+ \beta ^2_{ij} I^V_j)S_i - \frac{U_i(t) S_i}{N_i-W_i(t)}, \end{aligned}$$

2 $$\begin{aligned} \frac{dI_i}{dt}&= \sum _{j=1}^n(\beta ^1_{ij} I_j+ \beta ^2_{ij} I^V_j)S_i - \mu ^1_i I_i,\nonumber \\ \frac{dR_i}{dt}&= \mu ^1_i I_i,\nonumber \\ \frac{dS^V_i}{dt}&= -\sum _{j=1}^n(\beta ^3_{ij}I_j + \beta _{ij}^4 I^V_j)S^V_i + \frac{U_i(t) S_i}{N_i-W_i(t)},\nonumber \\ \frac{dI^V_i}{dt}&= \sum _{j=1}^n(\beta ^3_{ij}I_j + \beta _{ij}^4I^V_j)S^V_i -\mu ^2_i I^V_i, \nonumber \\ \frac{dR^V_i}{dt}&= \mu ^2_i I^V_i, \end{aligned}$$

where

$$\begin{aligned} W_i(t):= \int _0^t U_i(s)ds. \end{aligned}$$

Here, $\beta ^{1}_{ij}$ represents transmission from the unvaccinated members of group *j* to the unvaccinated members of group *i*, $\beta ^{2}_{ij}$ represents transmission from vaccinated members to unvaccinated members, $\beta ^{3}_{ij}$ represents transmission from vaccinated members to unvaccinated members and $\beta ^{4}_{ij}$ represents transmission from vaccinated members to vaccinated members. Additionally, $\mu _i^{1}$ represents the infectious period of unvaccinated infected members in group *i* while $\mu _i^2$ represents the infectious period of vaccinated members. Note that the superscript denotes different parameter values, so that $\beta ^2_{ij}$ is not necessarily the square of $\beta ^1_{ij}$.

To ensure that vaccination is “locally effective” (that is, a vaccinated individual is no more likely to transmit or be infected by the disease, and is infectious for no longer than an unvaccinated individual in the same subgroup), and that the parameters are epidemiologically feasible, the following constraints are imposed:

$$\begin{aligned} \beta ^1_{ij} \ge \beta _{ij}^2, \beta _{ij}^3 \ge \beta _{ij}^4\ge 0 \quad \text {and} \quad \mu _i^2 \ge \mu _i^1 > 0 \end{aligned}$$

Note that there is no constraint on the ordering of $\beta _{ij}^2$ and $\beta _{ij}^3$. It is assumed for convenience that all variables except the $S_i$ and $I_i$ are initially zero. Finally, we assume that all initial conditions are non-negative.

Ultimately, the objective of the vaccination program will be to minimize a weighted sum of the total infections in each group—that is

$$\begin{aligned} \sum _{i=1}^np_i(R_i(\infty ) + \kappa _iR_i^V(\infty )). \end{aligned}$$

Here $p_i$ is the weighting of a member of group *i* who is infected before being vaccinated, while $p_i\kappa _i$ is the weighting of a member of group *i* who is infected after being vaccinated. These parameters could be chosen to capture one of a range of objectives, such as minimizing deaths, minimizing hospitalisations, or minimizing total infections. Again assuming “local effectiveness” of the vaccination, it is imposed that $\kappa _i \le 1$, as vaccination should not increase the severity of the infection.

The equations (1)–(2) sum to zero on the right-hand side, and so for each *i*,

3 $$\begin{aligned} S_i(t) + I_i(t) + R_i(t) + S^V_i(t) + I^V_i(t) + R^V_i(t) = N_i \quad \forall t \ge 0. \end{aligned}$$

It will be assumed that the populations and parameters have been scaled such that $N = 1$, Finally, it is assumed that

$$\begin{aligned} W_i(t)\le N_i \quad \forall t \ge 0 \quad \text {and} \quad W_i(t) = N_i \Rightarrow \frac{U_i(t) S_i}{N_i-W_i(t)} = 0. \end{aligned}$$

to ensure feasibility of the vaccination policies.

### Comparison to the Standard Vaccination Model

A more common model of vaccination in the literature is the “standard” vaccination model (Hansen and Day 2011; Zaman et al. 2008; Kar and Batabyal 2011), where Eq. (1) becomes

$$\begin{aligned} \frac{dS_i}{dt} = -\sum _{j=1}^n(\beta ^1_{ij}I_j+ \beta ^2_{ij} I^V_j)S_i - U^*_i(t)S_i, \end{aligned}$$

Here, $U^*_i(t)$ is the vaccination rate in this model. In general, $U_i^*(t)$ is constrained such that $U_i^*(t) \le {\mathcal {U}}_i(t)$ for some function ${\mathcal {U}}_i(t)$

The $U^*_i(t)S_i$ term seeks to capture the fact that vaccination uptake will decrease even if the vaccination “effort” (or, equivalently, the doses available) remains constant. However, the rate at which uptake decreases is fixed by the model. For example, if the vaccination effort $U_i^*(t)$ was equal to a constant ${\mathcal {U}}_i$ and was much quicker than the rate of infection, then the leading order equation is

$$\begin{aligned} \frac{dS_i}{dt} = -{\mathcal {U}}_iS_i \Rightarrow S_i(t) = S_i(0)e^{-{\mathcal {U}}_i t} \end{aligned}$$

and hence

$$\begin{aligned} \frac{dS_i}{dt} = -{\mathcal {U}}_iS_i(0)e^{-{\mathcal {U}}_i t} \end{aligned}$$

which means that vaccination uptake decreases exponentially. Even for some human pandemics, such as COVID-19, where demand remained high until a large proportion of the population had been vaccinated, as shown in Ritchie et al. (2020), such a model may be inappropriate.

The general vaccination model provides substantially more flexibility. For example, it is possible for a group to be completely vaccinated in the general case, whereas this is impossible in the standard case (while one may never be able to fully vaccinate a human population, it would be possible, for example, in a group of animals on a farm). Moreover, by bounding the vaccination rate $U_i(t)$ above by some function of vaccination demand $K_i(W(t),t)$, decreasing vaccination uptake can still be modelled.

### Recovery of the Standard Model

The standard model is a special case of the general model, meaning that the results of this paper are applicable to both frameworks. To show this, one can solve the equation

4 $$\begin{aligned} \frac{U_i(t)}{N_i - W_i(t)} = U_i^*(t) \Rightarrow \frac{d}{dt}\bigg (\log (N_i-W_i(t)) + W_i^*(t)\bigg ) = 0, \end{aligned}$$

where

$$\begin{aligned} W^*_i(t):= \int _0^tU_i^*(s)ds. \end{aligned}$$

Thus, by integrating (4), and noting that $W^*_i(0) = W_i(0) = 0$

$$\begin{aligned} \log (N_i - W_i(t)) + W_i^*(t) = \log (N_i) \end{aligned}$$

and so

$$\begin{aligned} W_i(t) = N_i(1-e^{-W_i^*(t)}). \end{aligned}$$

The constraint $U^*_i(t) \le {\mathcal {U}}_i$ is equivalent to $U_i(t) \le (N_i-W_i(t)){\mathcal {U}}_i$ and so this can also be represented in the general model. Thus, given any standard vaccination policy ${\varvec{U}}^*$, it can be replaced by a general policy ${\varvec{U}}$ (although the converse does not hold as $W_i(t) = N_i$ requires $W_i^*(t) = \infty$).

Moreover, note that $W^*_i(t)$ is increasing in $W_i(t)$. Thus, if a pair of general policies ${\varvec{U}}$ and $\tilde{{\varvec{U}}}$ satisfy $W_i(t) \le {\tilde{W}}_i(t)$ then this inequality is preserved by the corresponding standard policies as $W^*_i(t) \le {\tilde{W}}^*_i(t)$. This property means that the theorems proved in this paper will hold for both models (as they will be proved using the general model).

## Optimisation Problem

Now that the model has been formulated, it is possible to set up the optimisation problem that will be considered in the remainder of this paper.

### Constraints on $U_i(t)$

In order to assist the proof of the theorems, it is necessary to make some (unrestrictive) assumptions on the vaccination rates, $U_i(t)$.

Firstly, there are the physical constraints that for each $i \in \{1,...,n\}$

5 $$\begin{aligned} U_i(t)\ge 0\quad \text {and} \quad \int _0^tU_i(s)ds \le N_i \quad \forall t \ge 0. \end{aligned}$$

It is also necessary that $U_i(t)$ is within the class of functions such that solutions to the model equations exist and are unique. Discussion of the exact conditions necessary for this to hold is outside the scope of this paper. However, from the Picard-Lindelöf Theorem (Collins 2006), a sufficient condition for this is that $U_i(t)$ is a piecewise Lipschitz continuous function. While this is not a necessary condition, this illustrates that this assumption will hold for a large class of functions. However, it will be helpful throughout the course of the proof to explicitly assume two conditions on $U_i(t)$ - namely, that it is bounded and that it is Lebesgue integrable on $\Re$ for each *i*.

For the remainder of this paper, define the set of feasible vaccination policies, *C*, is the set of functions ${\varvec{U}}$ satisfying (5) such that unique solutions to the model equations exist with these functions as the vaccination policy and such that each $U_i(t)$ is bounded and Lebesgue integrable on $\Re$.

### Optimisation Problem

The aim is to choose the vaccination policy ${\varvec{U}} \in C$ such that the total number of deaths (or any linear function of the infections in each subgroup) is minimised while meeting additional constraints on vaccine supply and vaccination rate. It is assumed that the maximal rate of vaccination at time *t* is *A*(*t*) and that there is a total (non-decreasing) supply of *B*(*t*) vaccinations that has arrived by time *t*. Thus, for each *i*, $U_i(t)$ is constrained to satisfy

$$\begin{aligned} \sum _{i=1}^nU_i(t) \le A(t)\quad \text {and} \quad \sum _{i=1}^n W_i(t) \le B(t). \end{aligned}$$

As previously discussed, it is assumed that each infection of unvaccinated people in group *i* is weighted by some $p_i$ and that the infection is no more serious for those that have been vaccinated, so that the weighting of an infection of a vaccinated person in group *i* is $p_i\kappa _i$, where $\kappa _i \le 1$. Thus, the objective function is

$$\begin{aligned} H({\varvec{U}}):= \sum _{i=1}^np_i\bigg (R_i(\infty ) + \kappa _i R^V_i(\infty )\bigg ) \end{aligned}$$

where, for example

$$\begin{aligned} R_i(\infty ) = \lim _{t \rightarrow \infty }(R_i(t)). \end{aligned}$$

Note these limits exist as $R_i$ is non-decreasing and bounded by Lemma C.3. Hence, the optimisation problem is

6 $$\begin{aligned} \min \bigg \{\sum _{i=1}^np_i\bigg (R_i(\infty ) + \kappa _i R^V_i(\infty )\bigg )&: \sum _{i=1}^nU_i(t) \le A(t),\quad \sum _{i=1}^n W_i(t) \le B(t) \quad \forall i,t...\nonumber \\&\text {and} \quad {\varvec{U}} \in C\bigg \}. \end{aligned}$$

## Main Results

The main results of this paper are as follows. Firstly, it is shown that the objective function is non-increasing in vaccination effort.

### Theorem 1

Suppose that ${\varvec{U}},\tilde{{\varvec{U}}} \in C$. Suppose further that for each $i \in \{1,...,n\}$ and $t \ge 0$

$$\begin{aligned} \int _0^t U_i(s)ds \le \int _0^t {\tilde{U}}_i(s)ds \end{aligned}$$

Then

$$\begin{aligned} H({\varvec{U}}) \ge H(\tilde{{\varvec{U}}}). \end{aligned}$$

Then, it is shown that if an optimal solution exists, there is an optimal maximal effort solution.

### Theorem 2

Suppose that *B* is differentiable, and that there is an optimal solution ${\varvec{U}}$ to (6). Then, define the function

$$\begin{aligned} \chi (t):= \left\{ \begin{matrix} A(t) &{} \text {if} \quad \int _0^t\chi (s)ds < B(t) \\ \min (A(t),B'(t)) &{} \text {if} \quad \int _0^t\chi (s)ds \ge B(t) \end{matrix}\right. \end{aligned}$$

and suppose that $\chi (t)$ exists and is bounded. Then, there exists an optimal solution $\tilde{{\varvec{U}}}$ to the problem (6) such that

7 $$\begin{aligned} \sum _{i=1}^n{\tilde{W}}_i(t) =\min \bigg (\int _0^t \chi (s)ds,1\bigg ). \end{aligned}$$

Moreover, if $\chi (t)$ is continuous almost everywhere, there exists an optimal solution $\tilde{{\varvec{U}}}$ such that

$$\begin{aligned} \sum _{i=1}^n {\tilde{U}}_i(t) = \left\{ \begin{matrix} \chi (t) &{} \text {if } \int _0^t \chi (s)ds < 1 \\ 0 &{} \text {otherwise}\end{matrix}\right. \end{aligned}$$

It is perhaps concerning to the reader that the existence of $\chi$ is left as an assumption in this theorem. However, while the exact conditions on its existence are beyond the scope of this paper, it certainly exists for a wide class of functions *A*(*t*) and *B*(*t*), as proved in Lemma B.11.

Finally, it is shown that this principle still holds if the cost of vaccination is considered.

### Theorem 3

Under the assumptions of Theorem 2, consider a modified objective function ${\mathcal {H}}$ given by

$$\begin{aligned} {\mathcal {H}}({\varvec{U}}) = H({\varvec{U}}) + F({\varvec{W}}(\infty )) \end{aligned}$$

for any function *F*. Then, with $\chi$ defined to be the maximal vaccination effort as in Theorem 2, there exists an optimal solution $\tilde{{\varvec{U}}}$ such that, for some $\tau \ge 0$

$$\begin{aligned} \sum _{i=1}^n{\tilde{W}}_i(t) =\left\{ \begin{matrix} \int _0^t \chi (s)ds&{} \text {if } t \le \tau \\ \\ W_i(\tau ) &{} \text {otherwise} \\ \end{matrix}\right. . \end{aligned}$$

Moreover, if $\chi$ is continuous almost everywhere, then there is an optimal solution $\tilde{{\varvec{U}}}$ such that

$$\begin{aligned} \sum _{i=1}^nU_i(t) =\left\{ \begin{matrix} \chi (t)&{} \text {if } t \le \tau \\ 0 &{} \text {otherwise} \\ \end{matrix}\right. . \end{aligned}$$

## Sketch Proof

The full proofs of Theorems 1, 2 and 3 can be found in Appendix A, with supplementary lemmas found in Appendix B and C. However, this section provides a high-level sketch of the main arguments.

### Bounds on the Inter-Group Infectious Forces

Define

$$\begin{aligned} K_{ij}(t) = \frac{\beta ^1_{ij}}{\mu ^1_j}R_j(t) + \frac{\beta ^2_{ij}}{\mu ^2_j}R^V_j(t) \end{aligned}$$

and

$$\begin{aligned} L_{ij}(t):= \frac{\beta _{ij}^3}{\mu _i^1} R_j(t) + \frac{\beta _{ij}^4}{\mu _i^2}R^V_j(t). \end{aligned}$$

$K_{ij}(t)$ can be interpreted as the total infectious force up to time *t* from the members of group *j* on the unvaccinated members of group *i* as

$$\begin{aligned} K_{ij}(t) = \int _0^t (\beta ^1_{ij}I_j({\tilde{t}}) + \beta ^2_{ij}I_j^V({\tilde{t}}))d{\tilde{t}}. \end{aligned}$$

Similarly, $L_{ij}(t)$ can be interpreted as the total infectious force up to time *t* from the members of group *j* on the vaccinated members of group *i*.

The first part of the proof shows that increasing the vaccination effort will decrease these infectious forces. To facilitate the proof, some extra assumptions are made on the parameters (which will be removed in subsequent propositions).

#### Proposition 1

Suppose that $U_i(t)$ and ${\tilde{U}}_i(t)$ are right-continuous step functions. Moreover, suppose that

$$\begin{aligned}{} & {} \beta ^1_{ij}> \beta _{ij}^3> 0 \quad \forall i,j \in \{1,...,n\}, \\{} & {} S_i(0)I_i(0) > 0 \quad \forall i \in \{1,...n\}. \end{aligned}$$

and that

$$\begin{aligned} W_i(t) < N_i \quad \forall t \ge 0 \quad \text {and} \quad \forall i \in \{1,...,n\} \end{aligned}$$

Then,

8 $$\begin{aligned} K_{ij}(t) \ge {\tilde{K}}_{ij}(t) \quad \text {and} \quad L_{ij}(t) \ge {\tilde{L}}_{ij}(t) \quad \forall t \ge 0. \end{aligned}$$

This proposition is proved by contradiction in two parts. Firstly, a time *T* is introduced, which is the infimum of the times where at least one of $K_{ij}(t)< {\tilde{K}}_{ij}(t)$ or $L_{ij}(t) < {\tilde{L}}_{ij}(t)$ for some *i* and *j*. As the infectious forces do not satisfy this condition in [0, *T*], one can show that, necessarily, they must all have been equal in [0, *T*], which means that one must have $W_i(t) = {\tilde{W}}_i(t)$ for all $t \in [0,T]$.

From here, the proof can proceed by a short-time linearisation, considering the small interval $[T,T+\delta ]$. The condition on $U_i$ and ${\tilde{U}}_i$ being step functions allows for them to be considered constant in this interval. It can then be shown that (8) must hold in $[T,T+\delta ]$, which contradicts the definition of *T* and completes the proof.

### A Proof for a Restricted Parameter and Policy Set

Proposition 1 can be extended to prove the result of Theorem 1 under the more restrictive set of conditions it introduced.

#### Proposition 2

Under the conditions of Proposition 1, for any $t \ge 0$ and $i \in \{1,...,n\}$

$$\begin{aligned} I_i(t) + I^V_i(t) + R_i(t) + R^V_i(t) \ge {\tilde{I}}_i(t) + {\tilde{I}}_i^V(t) +{\tilde{R}}_i(t) +{\tilde{R}}_i^V(t) \end{aligned}$$

and

$$\begin{aligned} R_i(t) \ge {\tilde{R}}_i(t). \end{aligned}$$

Moreover, for any $\lambda \in [0,1]$

$$\begin{aligned} R_i(\infty ) + \lambda R^V_i(\infty ) \ge {\tilde{R}}_i(\infty ) + \lambda {\tilde{R}}_i^V(\infty ) \end{aligned}$$

and hence, the objective function is lower for ${\tilde{U}}$, provided the conditions of Proposition 1 are met.

This comes from finding $S_i + S_i^V$ in terms of $K_{ij}$, $L_{ij}$ and *W*, and showing that $S_i + S_i^V \le {\tilde{S}}_i + {\tilde{S}}_i^V$—that is, that more people were infected in the $U_i$ case. Taking limits, and using a similar approach to consider the number of unvaccinated infections then shows the required result.

### Generalisation

This result can be generalised to the original set of parameters and vaccination policies by using the continuous dependence of the number of infections on the parameters and the vaccination policy.

From here, it is simple to weaken the inequalities on the parameters introduced in Proposition 1. The treatment of the vaccination policies requires more care, as it is not necessarily true that a Lebesgue intergrable ${\varvec{U}}$ can be approximated by step functions. However, its integral, ${\varvec{W}}$, can be approximated by the integral of step functions, and this allows the result of Proposition 2 to be generalised to Theorem 1.

### Theorem 2

Theorem 2 is proved as follows. Firstly, one can show that, for any vaccination policy ${\varvec{U}}$ and $t \ge 0$,

$$\begin{aligned} \min \bigg (\int _0^t\chi (s)ds,1\bigg ) \ge \int _0^t\sum _{i=1}^nU_i(s)ds, \end{aligned}$$

using the definition of $\chi$ in terms of the constraints on ${\varvec{U}}$. This means that the total rate of vaccination given by $\tilde{{\varvec{U}}}$ is at least as high as that given by ${\varvec{U}}$.

One can then show that $\chi (t) \le A(t)$

$$\begin{aligned} \int _0^t\chi (s)ds\le B(t) \end{aligned}$$

which means that $\tilde{{\varvec{U}}}$ satisfies the vaccination constraints.

From here, one can transform any optimal vaccination policy ${\varvec{U}}$ into suitable $\tilde{{\varvec{U}}}$. Initially, the quantities ${\tilde{W}}_i(t)$ are constructed. The details of this are left to the appendix but the general principle is that the policy ${\varvec{U}}$ is compressed in time so that the total number of vaccinations given out matches $\min \bigg (\int _0^t\chi (s)ds,1\bigg )$. It may also be necessary to add additional vaccinations if the overall total differs—these can be assigned in proportion to the number of unvaccinated people in each group.

This construction ensures that the feasibility constraints ${\tilde{W}}_i \le N_i$ are satisfied. Moreover, one can show that ${\tilde{W}}_i$ is Lipschitz continuous, which allows for the construction of a derivative ${\tilde{U}}_i$ which integrates to ${\tilde{W}}_i$. Finally, one can show that ${\tilde{W}}_i(t) \ge W_i(t)$, meaning that, by Theorem 1, $\tilde{{\varvec{U}}}$ must also be an optimal vaccination policy.

### Theorem 3

The proof of Theorem 3 then follows from a similar construction to Theorem 2—the only difference is that no additional vaccinations are assigned by $\tilde{{\varvec{U}}}$ compared to ${\varvec{U}}$.

## Limitations of Theorem 1

It is helpful to consider the limitations of Theorem 1, as it does not prove that every conceivable cost function is non-increasing in vaccination effort. This will be illustrated through some examples based on theoretical COVID-19 outbreaks in the United Kingdom.

Using the work of Prem et al. (2017), one can split the UK into 16 age-groups (comprising five-year intervals from 0 to 75 and a group for those aged 75+) which mix heterogeneously. The contact matrices estimated in Prem et al. (2017) allow for the construction of a matrix $\varvec{\beta }^*$, which will be proportional to each of the matrices $\varvec{\beta }^{\alpha }$ in the model.

As illustrated in Liu et al. (2020), estimation of the basic reproduction number $R_0$ for COVID-19 is complicated, and a wide range of estimates have been produced. For the examples in this paper, a reproduction number of 4 will be used, meaning that $\beta ^1$ will be scaled so that the largest eigenvalue of the matrix given by

$$\begin{aligned} M_{ij} = \frac{\beta ^1_{ij}N_i}{\mu _i^1} \end{aligned}$$

is equal to 4. Note that the population of each group ${\varvec{N}}$—normalised to have total sum 1—is taken from (UN 2019). Moreover, based on the estimates in Ram and Schaposnik (2021), the value of $\mu _i^1$ and, in the first example, $\mu _i^2$ will be set equal to $\frac{1}{14}$.

To model the effectiveness of vaccination, the estimates of Dean and Halloran (2022) will be used so that $\beta ^2 = 0.77\beta ^1$, (modelling the reduction in infectiousness), $\beta ^3 = 0.3\beta ^1$ (modelling the reduction in susceptibility) and $\beta ^4 = 0.77\times 0.3\times \beta ^1$ (assuming these effects are independent). Finally, the initial conditions used are $S_i(0) = (1-10^{-4})N_i$ and $I_i(0) = (10^{-4})N_i$ for each *i*, modelling a case where $0.01\%$ of the population is initially infected. It should be emphasised however, that this model has purely been made for illustrative purposes and substantially more detailed fitting analysis would be required to use it for forecasting COVID-19 in the UK.

In both the subsequent examples, it will be assumed that $0.5\%$ of the population is vaccinated homogeneously each day in the vaccination case. This will be compared to a case with no vaccination.

### Infections Are Not Decreasing For All Time

**Fig. 1:**
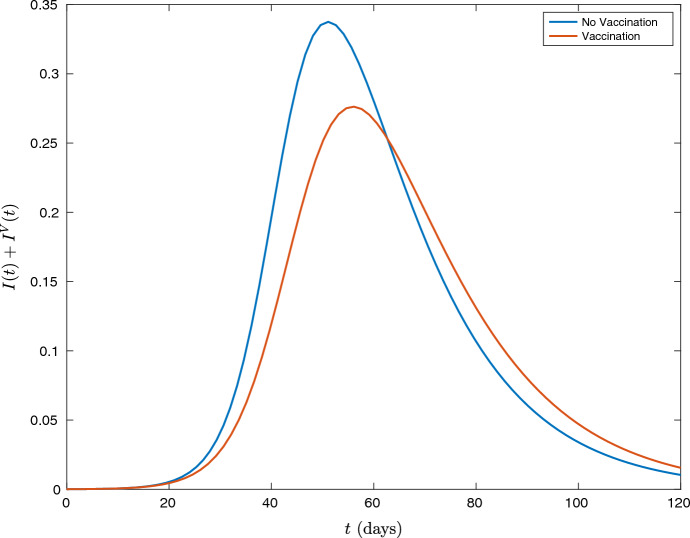
A comparison of the total infections over time for a simulated COVID-19 epidemic in the UK, depending on whether a uniform vaccination strategy of constant rate is used

While the overall number of infections will decrease as vaccination effort increases, the infections at a particular point in time will not. Figure 1 shows that the effect of vaccination is both to reduce, but also delay the peak of the infections. This is an important consideration when deciding vaccination policy, as increasing infections at a time in the year when hospitals are under more pressure could have negative consequences, and so it is important not to simply assume that vaccination will reduce all infections at all times.

### Deaths Are Not Decreasing For All Time

Perhaps most surprisingly, the total deaths in the epidemic may at some finite times (although not at $t = \infty$) be higher when vaccination occurs, at least under the assumptions of the SIR model. This is a rarer phenomenon, but is possible if vaccination increases the recovery rate as well as decreasing infectiousness.

**Fig. 2:**
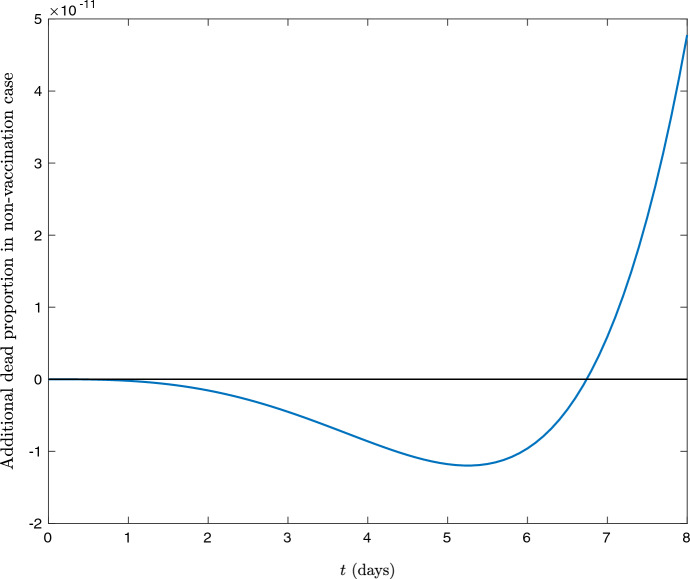
The difference between proportion of the population that has died by each time *t* in the case of vaccination and non-vaccination. Positive values indicate that the deaths are higher in the non-vaccination case

For illustrative purposes, suppose that vaccination doubles the recovery rate (so that $\mu _i^2 = \frac{1}{7}$) and has no effect on mortality rates. Then, using Bonanad et al. (2020) to get age-dependent mortality rates for COVID-19, Fig. 2 shows that initially, the number of deaths is higher in the case of vaccination. This occurs because the higher value of $\mu ^2$ means that vaccinated people move more quickly to the $R^V$ compartment than their unvaccinated counterparts and so, while they will infect fewer people, when the number of infections is comparable in the early epidemic, this means that more people will die. Indeed, this property can still hold if vaccination reduced mortality rates (although this reduces the already small difference between the two further—in this example, one needs $\kappa _i \gtrsim 0.9999$ for deaths to ever be lower in the non-vaccinated case).

Of course, this is not a realistic reflection of the course of an epidemic—the reason for $\mu ^2$ being higher is that vaccinated people are likely to get *less* ill rather than dying more quickly—but it illustrates a potential limitation of the SIR framework. One possible way to avoid this problem would be to split the recovered compartment up into the truly recovered and dead subsections. Then, vaccination could increase the speed at which infected members of the population moved to the recovered compartment, but not the speed at which they moved to the dead compartment. This would remove the possibility of seeing the counter-intuitive behaviour of Fig. 2.

## Discussion

It is comforting that the multi-group SIR model does indeed satisfy the condition that the final numbers of infections and deaths are non-increasing in vaccination effort. This shows the importance of ensuring that vaccinations are available as early as possible in a disease outbreak. To achieve this, it is important that good plans for vaccine roll-out and supply chains are available in advance of them being needed to ensure that maximum benefit from the vaccination program is obtained.

For $n > 1$, there are, of course, many possible maximal-effort vaccination policies. The results of this paper, in effect, reduce the dimension of the space of possible vaccination policies from *n* to $n-1$, as one can assume that an optimal policy satisfies the condition (7) in Theorem 2. However, choosing the correct groups to prioritise is still of crucial importance and can have a substantial impact of the effectiveness of the vaccination campaign (Fitzpatrick and Galvani 2021). Applying similarly rigorous techniques to finding the optimal vaccination policy is beyond the scope of this paper, although we extended the results of this paper to apply asymptotic techniques to understand the behaviour of the optimal solution under certain special cases in Penn and Donnelly (2023).

However, there are limitations to these results. Indeed, while the final numbers of infections and deaths are guaranteed to decrease, this is not necessarily true at a given finite time. In particular, vaccination can move the peak of the epidemic, and so it is important to consider the consequences of this, particularly if only a small number of lives are saved by vaccination.

Moreover, while this has not been discussed in this paper, it is also important to emphasise that these results only apply if vaccine efficacy does not decay over time. Indeed, if vaccination efficacy does decay significantly, then vaccinating the most vulnerable groups in a population very early may be worse than vaccinating them later, unless booster jabs are available. If the main epidemic occurs long after the vulnerable have been vaccinated, their immunity may have worn off significantly by the time that the majority of disease exposure occurs. Thus, in this case a more detailed analysis would be needed to determine the optimal vaccination rate.

The authors believe that future models for optimal vaccination should consider using the more general vaccination model introduced in this paper. This allows for greater flexibility in modelling the effect of decreasing demand. Of course, this modified model is slightly more complicated, and care needs to be taken to avoid numerical instabilities arising from the removable singularity in the $\frac{U_i S_i}{N_i - W_i}$ term when $W_i \rightarrow N_i$. However, it has been shown that many of the standard properties of SIR models, and indeed the results of this paper, still hold for this model, and so these extra technical difficulties appear to be a small price to pay for the significantly increased accuracy and potentially large difference between the optimal solutions for the two models.

The results of this paper could be extended to cover a wider range of disease models that are currently being used in the literature. In particular, the next step could be to prove the results for SEIR (Susceptible-Exposed-Infected-Recovered) models, and indeed models with multiple exposed compartments for each subgroup. This would help to build a general mathematical theory of maximal-effort vaccination that would provide evidence for the reliability of contemporary epidemiological modelling.

## Conclusion

The results of this paper are summarised below:

- Vaccinating at maximal effort is optimal for a multi-group SIR model with non-decaying vaccination efficacy.
- The general vaccination model introduced in this paper provides greater flexibility in modelling the effect of decreasing vaccination uptake.
- While vaccinating at maximal effort gives optimality, there can be finite times at which, according to the SIR model, infections or deaths are higher if vaccination has occurred.

## Data Availability

As cited in the text, the data used to create Fig. 1 is available from https://journals.plos.org/ploscompbiol/article?id=10.1371/journal.pcbi.1005697 and https://population.un.org/wpp/Download/Standard/Population/. The additional data used to create Fig. 2 is available from https://www.sciencedirect.com/science/article/pii/S1525861020304412.

## Proofs of Theorems 1, 2 and 3

Before beginning the main proof, it is helpful to note some fundamental results about the SIR equations that will be used throughout. Namely, for each $i\in \{1,...,n\}$ and $t \ge 0$

$$\begin{aligned} 0 \le S_i(t), I_i(t), R_i(t), S^V_i(t), I^V_i(t), R_i^V(t) \le N_i \end{aligned}$$

and

$$\begin{aligned} \lim _{t \rightarrow \infty }(I_i(t)) = \lim _{t \rightarrow \infty }(I^V_i(t)) = 0. \end{aligned}$$

These results are proved in Lemmas C.3 and C.4.

It is first useful to define

$$\begin{aligned} K_{ij}(t) = \frac{\beta ^1_{ij}}{\mu ^1_j}R_j(t) + \frac{\beta ^2_{ij}}{\mu ^2_j}R^V_j(t) \end{aligned}$$

and

$$\begin{aligned} L_{ij}(t):= \frac{\beta _{ij}^3}{\mu _i^1} R_j(t) + \frac{\beta _{ij}^4}{\mu _i^2}R^V_j(t). \end{aligned}$$

Then, the following propositions hold.

### An Inequality for $K_{ij}$ and $L_{ij}$

Note that the proof of this proposition requires a significant amount of algebra, and the majority of it has hence been left to lemmas which can be found in Appendix B. However, the key logic of the proof will be presented here.

Also, note that in this paper, a step function is defined to be a function that is piecewise constant on any *bounded* interval of $\Re$. Thus, it may have infinitely many discontinuities, but only finitely many in any bounded interval. This differs from the definition used in some other papers (which impose that a step function is piecewise constant on $\Re$).

#### Proposition A.1.1

Suppose that $U_i(t)$ and ${\tilde{U}}_i(t)$ are right-continuous step functions. Moreover, suppose that

$$\begin{aligned}{} & {} \beta ^1_{ij}> \beta _{ij}^3> 0 \quad \forall i,j \in \{1,...,n\}, \\{} & {} \quad _i(0)I_i(0) > 0 \quad \forall i \in \{1,...n\}. \end{aligned}$$

and that

$$\begin{aligned} W_i(t) < N_i \quad \forall t \ge 0 \quad \text {and} \quad \forall i \in \{1,...,n\} \end{aligned}$$

Then,

$$\begin{aligned} K_{ij}(t) \ge {\tilde{K}}_{ij}(t) \quad \text {and} \quad L_{ij}(t) \ge {\tilde{L}}_{ij}(t) \quad \forall t \ge 0. \end{aligned}$$

#### Proof

Suppose that the proposition does not hold. Hence, one can define

$$\begin{aligned} T:= \inf \left\{ t: K_{ij}(t)<{\tilde{K}}_{ij}(t) \quad \text {or} \quad L_{ij}(t) < {\tilde{L}}_{ij}(t) \quad \text {for some } i,j \in \{1,...,n\} \right\} . \end{aligned}$$

Then, there exists some $b \in \{1,..,n\}$ and some real constants $\kappa$ and $\eta$ such that the following system of inequalities holds at time *T*:

9 $$\begin{aligned} S_b(T)+S^V_b(T)&\le {\tilde{S}}_b(T) + {\tilde{S}}^V_b(T), \end{aligned}$$

10 $$\begin{aligned} I_b(T) + R_b(T)&\ge {\tilde{I}}_b(T) + {\tilde{R}}_b(T) \end{aligned}$$

11 $$\begin{aligned} R_b(T)&\ge {\tilde{R}}_b(T), \end{aligned}$$

12 $$\begin{aligned} R_b(T) + \kappa R^V_b(T)&\le {\tilde{R}}_b(T) + \kappa {\tilde{R}}^V_b(T), \end{aligned}$$

13 $$\begin{aligned} I_b(T) + \eta I^V_b(T)&\le {\tilde{I}}_b(T) + \eta {\tilde{I}}^V_b(T), \end{aligned}$$

14 $$\begin{aligned} 0 \le \kappa&\le \eta \le 1. \end{aligned}$$

The derivations of inequalities (9) - (14) are found in Lemmas B.2–B.5. Moreover,

15 $$\begin{aligned}&S_b(T) + I_b(T) + R_b(T) + S^V_b(T) + I^V_b(T) + R^V_b(T) \nonumber \\&\quad = {\tilde{S}}_b(T) + {\tilde{I}}_b(T) + {\tilde{R}}_b(T) + {\tilde{S}}^V_b(T)+ {\tilde{I}}^V_b(T) + {\tilde{R}}^V_b(T), \end{aligned}$$

which comes from (3). Note that (12) in fact holds to equality in this case, but this is not necessary for the proof (and later, the same system will be considered where such an equality is not guaranteed).

By Lemma B.6, the system (9)–(15) implies that

16 $$\begin{aligned} I_b(T) + R_b(T)&= {\tilde{I}}_b(T) + {\tilde{R}}_b(T), \end{aligned}$$

17 $$\begin{aligned} I_b^V(T) + R_b^V(T)&= {\tilde{I}}^V_b(T) + {\tilde{R}}^V_b(T), \end{aligned}$$

18 $$\begin{aligned} S_b(T) + S_b^V(T)&= {\tilde{S}}_b(T) + {\tilde{S}}_b^V(T), \end{aligned}$$

If $T > 0$, then Lemma B.7 can be used to show that

$$\begin{aligned} W_k(t) = {\tilde{W}}_k(t) \quad \forall t \in [0,T] \quad \text {and}\quad \forall k \in \{1,...,n\} \end{aligned}$$

while if $T = 0$ then this is immediate. Thus, the two ODE systems are the same up to time *T*, which means that all variables (in all groups) are equal at time *T*.

From this point, the proof of Proposition A.1.1 can be completed by considering the behaviour of the system at time $T+\delta$ for small $\delta$. For sufficiently small $\delta$, $U_i(t)$ and ${\tilde{U}}_i(t)$ are constant on $[T,T+\delta ]$ (as they are step functions) and this condition on $\delta$ will be assumed for the remainder of this proof

Define functions $\Delta ^f_i$ to be

$$\begin{aligned} \Delta ^f_i(t):= f_i(T+t) - {\tilde{f}}_i(T+t) \quad \text {for} \quad f \in \{S,I,R,S^V,I^V,R^V,W\} \end{aligned}$$

and note that

$$\begin{aligned} \Delta ^f_i(0) = 0 \quad \forall f \in \{S,I,R,S^V,I^V,R^V,W\}. \end{aligned}$$

Then, by Lemma B.8, for $t \in [0,\delta ]$ and any real numbers *x* and *y*

$$\begin{aligned} \frac{x}{\mu _i^1}\Delta ^R_i + \frac{y}{\mu _i^2} \Delta ^{R^V}_i&= \frac{t^3S_i(T)(U_i(T)-{\tilde{U}}_i(T))}{6(N_i-W_i(T))}\left[ x\sum _{j=1}^n(K_{ij}'(T)) -y\sum _{j=1}^n(L'_{ij}(T))\right] + O(\delta ^4). \end{aligned}$$

Hence, by Lemma B.9,

19 $$\begin{aligned} \sum _{j=1}^nK_{ij}(t) \ge \sum _{j=1}^n{\tilde{K}}_{ij}(t) \quad \forall t \in [0,T+\delta ] \end{aligned}$$

and

20 $$\begin{aligned} \sum _{j=1}^nL_{ij}(t) \ge \sum _{j=1}^n{\tilde{L}}_{ij}(t) \quad \forall t \in [0,T+\delta ] \end{aligned}$$

for sufficiently small $\delta$.

Now, by the definition of *T*, there exists some *t* in $[T,T+\delta ]$ such that, for some *a*, *b*

$$\begin{aligned} K_{ab}(t)< {\tilde{K}}_{ab}(t) \quad \text {or} \quad L_{ab}(t) < {\tilde{L}}_{ab}(t). \end{aligned}$$

Indeed, from Lemma B.10, there exists some $t \in (T,T+\delta )$ such that

21 $$\begin{aligned} R_b(t) + \kappa R^V_b(t) < {\tilde{R}}_b(t) + \kappa {\tilde{R}}^V_b(t) \quad \text {and} \quad I_b(t) + \eta I^V_b(t) \le {\tilde{I}}_b(t) + \eta {\tilde{I}}^V_b(t)\nonumber \\ \end{aligned}$$

for some

22 $$\begin{aligned} 0 \le \kappa \le \eta \le 1. \end{aligned}$$

Now, by Lemmas B.2–B.4 (which only require the properties (19) and (20)), the system of inequalities (9)–(11) holds for group *b* at time *t*. These can be combined with (21), (22) and (15) to use Lemma B.6, showing

23 $$\begin{aligned} \eta I_b^V(t) + \kappa R_b^V(t)&= \eta {\tilde{I}}_b^V(t) + \kappa {\tilde{R}}_b^V(t) \end{aligned}$$

24 $$\begin{aligned} I_b(t) + R_b(t)&= {\tilde{I}}_b(t) + {\tilde{R}}_b(t). \end{aligned}$$

By adding the inequalities in (21) together,

$$\begin{aligned} R_b(t) + \kappa R^V_b(t) + I_b(t) + \eta I^V_b(t) < {\tilde{R}}_b(t) + \kappa {\tilde{R}}^V_b(t) + {\tilde{I}}_b(t) + \eta {\tilde{I}}^V_b(t). \end{aligned}$$

Then, (23) and (24) show that this must in fact be an equality which is a contradiction. Thus, *t* cannot exist. This provides a contradiction to the definition of *T*, and hence finishes the proof of Proposition A.1.1.

It is now possible to prove Theorem 1 under the extra restrictions given Proposition A.1.1. $\square$

### A Proof for a Restricted Parameter and Policy Set

#### Proposition A.2.1

Under the conditions of Proposition A.1.1, for any $t \ge 0$ and $i \in \{1,...,n\}$

$$\begin{aligned} I_i(t) + I^V_i(t) + R_i(t) + R^V_i(t) \ge {\tilde{I}}_i(t) + {\tilde{I}}_i^V(t) +{\tilde{R}}_i(t) +{\tilde{R}}_i^V(t) \end{aligned}$$

and

$$\begin{aligned} R_i(t) \ge {\tilde{R}}_i(t). \end{aligned}$$

Moreover, for any $\lambda \in [0,1]$

$$\begin{aligned} R_i(\infty ) + \lambda R^V_i(\infty ) \ge {\tilde{R}}_i(\infty ) + \lambda {\tilde{R}}_i^V(\infty ) \end{aligned}$$

and hence, the objective function is lower for ${\tilde{U}}$, provided the conditions of Proposition A.1.1 are met.

#### Proof

Note that, by Proposition A.1.1,

$$\begin{aligned} K_{ij}(t) \ge {\tilde{K}}_{ij}(t) \quad \text {and} \quad L_{ij}(t) \ge {\tilde{L}}_{ij}(t) \quad \forall t \ge 0 \end{aligned}$$

and hence, by Lemma B.2, for each $i \in \{1,...,n\}$

$$\begin{aligned} S_i(t) + S_i^V(t) \le {\tilde{S}}_i(t) + {\tilde{S}}^V_i(t). \end{aligned}$$

Combining this with the conservation of population Eq. (15), shows that

$$\begin{aligned} I_i(t) + I^V_i(t) + R_i(t) + R^V_i(t) \ge {\tilde{I}}_i(t) + {\tilde{I}}^V_i(t) + {\tilde{R}}_i(t) + {\tilde{R}}_i^V(t) \end{aligned}$$

as required. Now, taking $t \rightarrow \infty$ and noting that the infections tend to zero by Lemma C.4 gives

$$\begin{aligned} R_i(\infty ) + R^V_i(\infty ) \ge {\tilde{R}}_i(\infty ) + {\tilde{R}}_i^V(\infty ). \end{aligned}$$

Moreover, by Lemma B.4, for any $t \ge 0$ and $i \in \{1,...,n\}$

$$\begin{aligned} R_i(t)\ge {\tilde{R}}_i(t) \end{aligned}$$

as required. Also, taking $t \rightarrow \infty$ shows that

$$\begin{aligned} R_i(\infty )\ge {\tilde{R}}_i(\infty ). \end{aligned}$$

Thus, for any $\lambda \in [0,1]$

$$\begin{aligned} R_i(\infty ) + \lambda R_i^V(\infty )&= (1-\lambda )R_i(\infty ) + \lambda (R_i(\infty ) + R_i^V(\infty )) \\&\ge (1-\lambda ){\tilde{R}}_i(\infty ) + \lambda ({\tilde{R}}_i(\infty ) + {\tilde{R}}_i^V(\infty )) \\&= {\tilde{R}}_i(\infty ) + \lambda {\tilde{R}}_i^V(\infty ) \end{aligned}$$

as required. $$\square $$

By summing the *i* inequalities at $t=\infty$ from Proposition A.2.1 (and using $\lambda = \kappa _i$), Theorem 1 holds under the additional conditions given in Proposition A.1.1. Note that the closure of the set of parameters, initial conditions and vaccination policies which satisfy these conditions is the original set specified in Theorem 1. Thus, one can generalise the result with the help of the following proposition.

### Continuous Dependence

#### Proposition A.3.1

Define the set of functions

$$\begin{aligned} {\mathcal {F}}:= \bigg \{ S_i(t;\epsilon ), I_i(t;\epsilon ),R_i(t;\epsilon ),S^V_i(t;\epsilon ),I^V_i(t;\epsilon ),R^V_i(t;\epsilon ): i \in \{1,...,n\}, \quad \epsilon ,t \ge 0\bigg \}, \end{aligned}$$

where for each fixed $\epsilon$, these functions solve the model equations with parameters

$$\begin{aligned} {\mathcal {P}}= \bigg \{\beta _{ij}^{\alpha }(\epsilon ), \mu _i^{\gamma }(\epsilon ): i,j \in \{1,...,n\}, \quad \alpha \in \{1,2,3,4\}, \quad \gamma \in \{1,2\} \quad \text {and} \quad \epsilon \ge 0\bigg \}, \end{aligned}$$

initial conditions

$$\begin{aligned} {\mathcal {I}}= \bigg \{f(0;\epsilon ): i \in \{1,...,n\}, \quad f \in {\mathcal {F}} \quad \text {and} \quad \epsilon \ge 0\bigg \} \end{aligned}$$

and vaccination policy ${\varvec{U}}(t;\epsilon )$. Suppose that

$$\begin{aligned} \vert p(\epsilon ) - p(0)\vert\le & {} \epsilon \quad \forall p \in {\mathcal {P}}, \\ \vert f_i(0;\epsilon ) - f_i(0;0)\vert\le & {} \epsilon \quad \forall f \in {\mathcal {F}} \end{aligned}$$

and that

$$\begin{aligned} \vert W_i(t,\epsilon ) - W_i(t,0)\vert < \epsilon \quad \forall t \ge 0. \end{aligned}$$

Moreover, suppose that for each $i \in \{1,...,n\}$ and $\epsilon \ge 0$,

$$\begin{aligned} U_i(s;\epsilon ) \ge 0 \quad \text {and} \quad \int _0^t U_i(s;\epsilon ) ds \le N_i \quad \forall t \ge 0. \end{aligned}$$

Then, for each $\delta > 0$ and each $T>0$ there exists some $\eta > 0$ (that may depend on *T* and $\delta$) such that

$$\begin{aligned} \epsilon \in (0,\eta ) \Rightarrow \vert f(t;\epsilon ) - f(t;0)\vert < \delta \quad \forall f \in {\mathcal {F}} \quad \text {and} \quad \forall t \in [0,T] \end{aligned}$$

#### Proof

The proof is simple but algebraically dense and so is left to Lemma C.8 in the appendices.

This now allows a proof of Theorem 1 to be formed. $\square$

### Theorem 1

#### Theorem 1

Suppose that ${\varvec{U}},\tilde{{\varvec{U}}} \in C$. Suppose further that for each $i \in \{1,...,n\}$ and $t \ge 0$

$$\begin{aligned} \int _0^t U_i(s)ds \le \int _0^t {\tilde{U}}_i(s)ds. \end{aligned}$$

Then

$$\begin{aligned} H({\varvec{U}}) \ge H(\tilde{{\varvec{U}}}). \end{aligned}$$

#### Proof

Define the parameters $\beta ^a_{ij}(\epsilon )$ and $\mu ^a_i(\epsilon )$ by

$$\begin{aligned} \beta ^a_{ij}(\epsilon ) = \beta ^a_{ij} + \frac{\epsilon }{a} \quad \text {and} \quad \mu _i^a(\epsilon ) = \mu _i^a. \end{aligned}$$

This means that, for any $\epsilon > 0$, these parameters satisfy the conditions of Propositions A.1.1 and A.2.1. Define, for $\epsilon < 1$, the initial conditions

$$\begin{aligned} S_i(0;\epsilon ) = \left\{ \begin{matrix} S_i(0;0) &{} \text {if } S_i(0;0), I_i(0;0) > 0 \\ S_i(0;0) + \epsilon N_i &{} \text {if } S_i(0;0) = 0\\ S_i(0;0) - \epsilon N_i &{} \text {if } I_i(0;0) = 0 \\ \end{matrix}\right. \end{aligned}$$

and

$$\begin{aligned} I_i(0;\epsilon ) = N_i - S_i(0;\epsilon ). \end{aligned}$$

Then, the conditions of Propositions A.1.1 and A.2.1 are met by these initial conditions for any $\epsilon > 0$.

Now, define the set of points

$$\begin{aligned} \sigma (\epsilon ):= \bigg \{n\epsilon : n \in {\mathcal {N}}_{\ge 0}\bigg \}. \end{aligned}$$

Then, define $W_i^*(t;\epsilon )$ to be the first order approximation to the function ${\mathcal {W}}_i(t;\epsilon ):= \max (W_i(t),N_i-\epsilon )$ using the points of $\sigma (\epsilon )$. That is, for each *t* define

$$\begin{aligned} K(t;\epsilon ):=\inf \bigg \{m: m \in \sigma (\epsilon ) \quad \text {and} \quad m\ge t\bigg \} \end{aligned}$$

and

$$\begin{aligned} k(t;\epsilon ):= \sup \bigg \{m: m \in \sigma (\epsilon ) \quad \text {and} \quad m \le t\bigg \} \end{aligned}$$

Note that, as $\sigma (\epsilon )$ is nowhere dense, one must have

$$\begin{aligned} k(t;\epsilon ), K(t;\epsilon ) \in \sigma (\epsilon ) \quad \text {and} \quad k(t;\epsilon ) \le t \le K(t;\epsilon ) \end{aligned}$$

Then, define

$$\begin{aligned} W_i^*(t;\epsilon ) = (t - k(t;\epsilon )){\mathcal {W}}_i(k(t;\epsilon );\epsilon ) + (K(t;\epsilon ) - t){\mathcal {W}}_i(K(t;\epsilon );\epsilon ). \end{aligned}$$

Thus, as *k* and *K* are constant on any interval not containing a point in $\sigma (\epsilon )$, $W_i^*$ is linear on any interval not containing a point of $\sigma (\epsilon )$ and so its derivative is a step function. $\square$

Now, note that, for each *t*

$$\begin{aligned} \vert {\mathcal {W}}_i(t;\epsilon ) - W_i(t)\vert \le \epsilon \end{aligned}$$

and, moreover,

$$\begin{aligned} t \in S \Rightarrow W_i^*(t;\epsilon ) = {\mathcal {W}}_i(t;\epsilon ). \end{aligned}$$

Also, as $U_i$ is bounded, each $W_i$ (and hence each ${\mathcal {W}}_i$) are Lipschitz continuous with some Lipschitz constant *L*. Moreover, each $W_i^*$ is continuous and is differentiable in each interval $(k(t;\epsilon ),K(t;\epsilon ))$ with a maximal (uniformly bounded) gradient of $U_i(t)$, meaning that $W_i^*$ is also Lipschitz continuous with Lipschitz constant *L*.

It can now be shown that $\vert W_i(t) - W_i^*(t;\epsilon )\vert$ is uniformly bounded in *t*. For each $t\ge 0$, one can find an element $s\in \sigma (\epsilon )$ such that $\vert t-s\vert < \epsilon$. Then,

$$\begin{aligned} \vert W_i(t) - W_i^*(t;\epsilon )\vert&\le \vert W_i(t) - W_i(s)\vert + \vert W_i(s) - W_i^*(s;\epsilon )\vert + \vert W_i^*(s;\epsilon ) - W_i^*(t;\epsilon )\vert \\&\le L\epsilon + \vert W_i(s) - {\mathcal {W}}_i(s;\epsilon ) \vert + L\epsilon \\&\le (2L+1)\epsilon \end{aligned}$$

and so $W_i^*$ converges uniformly to $W_i$. The same results hold for the analogously defined ${\tilde{W}}_i^*$. Then, note that, as ${\tilde{W}}_i(t) \ge W_i(t)$, it must be that $\tilde{{\mathcal {W}}}_i(t;\epsilon )\ge {\mathcal {W}}_i(t;\epsilon )$. Thus, it follows that ${\tilde{W}}_i^*(t;\epsilon ) \ge W_i^*(t;\epsilon )$.

This means that Proposition A.2.1 can be used. Define using stars the variables that come from the ${\varvec{U}}^*$ and $\tilde{{\varvec{U}}}^*$ policies. Then, from Proposition 2, for each $t \ge 0$, $\epsilon > 0$ and $i \in \{1,...,n\}$

$$\begin{aligned}{} & {} I_i^*(t;\epsilon ) + I^{V}_{i}{}^*(t;\epsilon ) + R_i^*(t;\epsilon ) + R^{V}_{i}{}^*(t;\epsilon ) \ge {\tilde{I}}_i^*(t;\epsilon )\\ {}{} & {} + {\tilde{I}}^{V}_{i}{}^*(t;\epsilon ) + {\tilde{R}}_i^*(t;\epsilon ) + {\tilde{R}}_{i}^{V}{}^*(t;\epsilon ) \end{aligned}$$

and

$$\begin{aligned} R^*_i(t;\epsilon )\ge {\tilde{R}}^*_i(t;\epsilon ). \end{aligned}$$

Then, taking $\epsilon \rightarrow 0$ and using Proposition A.3.1 (noting that the perturbations to the parameters, initial conditions and vaccination policies are all bounded by a constant multiple of $\epsilon$) shows that

$$\begin{aligned} I_i(t) + I^V_i(t) + R_i(t) + R^V_i(t) \ge {\tilde{I}}_i(t) + {\tilde{I}}^V_i(t) + {\tilde{R}}_i(t) + {\tilde{R}}_i^V(t) \end{aligned}$$

and

$$\begin{aligned} R_i(t) \ge R^V_i(t). \end{aligned}$$

Then, the result follows using the same logic as in the proof of Proposition A.2.1.

### Theorem 2

#### Theorem 2

Suppose that *B* is differentiable, and that there is an optimal solution ${\varvec{U}}$ to (6). Then, define the function

$$\begin{aligned} \chi (t):= \left\{ \begin{matrix} A(t) &{} \text {if} \quad \int _0^t\chi (s)ds < B(t) \\ \min (A(t),B'(t)) &{} \text {if} \quad \int _0^t\chi (s)ds \ge B(t) \end{matrix}\right. \end{aligned}$$

and suppose that $\chi (t)$ exists and is bounded. Then, there exists an optimal solution $\tilde{{\varvec{U}}}$ to the problem (6) such that

$$\begin{aligned} \sum _{i=1}^n{\tilde{W}}_i(t) =\min \bigg (\int _0^t \chi (s)ds,1\bigg ). \end{aligned}$$

Moreover, if $\chi (t)$ is continuous almost everywhere, there exists an optimal solution $\tilde{{\varvec{U}}}$ such that

$$\begin{aligned} \sum _{i=1}^n {\tilde{U}}_i(t) = \left\{ \begin{matrix} \chi (t) &{} \text {if } \int _0^t \chi (s)ds < 1 \\ 0 &{} \text {otherwise}\end{matrix}\right. \end{aligned}$$

#### Proof

Suppose that ${\varvec{U}}$ is an optimal vaccination policy. To begin, it will be shown that the total vaccination rate $\chi$ is indeed a maximal-effort vaccination policy (in the sense that, at each time $t^*$, it is impossible to have given out more vaccines than a policy with total overall rate $\chi (t)$). $\square$

**Claim:** $\min \bigg (1,\int _0^{t}\chi (s)ds\bigg ) \ge \int _0^{t}\sum _{i=1}^nU_i(s)ds$ for all $t > 0$

#### Proof

Consider any time $t \ge 0$ such that

$$\begin{aligned} \int _0^{t}\chi (s)ds < 1 \end{aligned}$$

and define the set

$$\begin{aligned} {\mathcal {T}}:= \left\{ s \le t: \int _0^s\chi (k)dk \ge B(s) \right\} . \end{aligned}$$

Suppose that ${\mathcal {T}} = \emptyset$. Then,

$$\begin{aligned} \chi (s) = A(s) \quad \forall s \le t \end{aligned}$$

and so

$$\begin{aligned} \int _0^{t}\chi (s)ds = \int _0^{t}A(s)ds \ge \int _0^{t}\sum _{i=1}^nU_i(s)ds. \end{aligned}$$

Moreover, suppose that ${\mathcal {T}} \ne \emptyset$ and define

$$\begin{aligned} \tau := \sup ({\mathcal {T}}). \end{aligned}$$

Then,

$$\begin{aligned} \int _0^{\tau }\chi (s)ds \ge B(\tau ) \ge \int _0^{\tau }\sum _{i=1}^nU_i(s)ds \end{aligned}$$

and

$$\begin{aligned} \int _{\tau }^{t}\chi (s)ds = \int _{\tau }^{t}A(s)ds \ge \int _{\tau }^{t}\sum _{i=1}^nU_i(s)ds \end{aligned}$$

so that

$$\begin{aligned} \int _0^{t}\chi (s)ds \ge \int _0^{t}\sum _{i=1}^nU_i(s)ds. \end{aligned}$$

Thus, this holds in all cases for $\int _0^t\chi (s)ds <1$. Finally, suppose that

$$\begin{aligned} \int _0^{t}\chi (s)ds \ge 1. \end{aligned}$$

Then, one has

$$\begin{aligned} \min \bigg (1,\int _0^{t^*}\chi (s)ds\bigg ) = 1 = \sum _{i=1}^nN_i \ge \int _0^{t^*}\sum _{i=1}U_i(s)ds \end{aligned}$$

and so the claim is proved. $\square$

It is now important to show that $\chi$ gives a feasible vaccination rate. Note that $\chi (t) \le A(t)$ by definition.

**Claim:** $\int _0^t\chi (s)ds \le B(t)$ for all $t \ge 0$.

#### Proof

Suppose, for a contradiction, that there exists a *t* such that

$$\begin{aligned} \int _0^t\chi (s)ds > B(t). \end{aligned}$$

Then, define

$$\begin{aligned} \sigma := \sup \bigg \{ s \le t: \int _0^t\chi (s)ds \le B(t) \bigg \} \end{aligned}$$

which must exist (as $\int _0^0\chi (s)ds \le B(0)$) and satisfy $\sigma < t$, by continuity of $\int _0^t\chi (s)ds$ and *B*(*t*). Note that

$$\begin{aligned} s \in (\sigma ,t) \Rightarrow \chi (s) \le B'(s) \end{aligned}$$

and so

$$\begin{aligned} \int _0^t\chi (s)ds \le \int _0^{\sigma }\chi (s)ds + \int _{\sigma }^t B'(s)ds \le B(\sigma ) + (B(t) - B(\sigma )) = B(t), \end{aligned}$$

which is a contradiction. Thus,

$$\begin{aligned} \int _0^t\chi (s)ds \le B(t) \quad \forall t \ge 0 \end{aligned}$$

as required. $$\square $$

Now, one can create a new optimal vaccination policy with total rate given by $\chi$. Define

$$\begin{aligned} q(t) = \left\{ \begin{matrix} \inf \bigg \{ s: \int _0^s \sum _{j=1}^n U_j(k)dk = \int _0^t \chi (k)dk\bigg \} &{} \text {if this exists} \\ \\ \infty &{} \text {otherwise}\end{matrix}\right. \end{aligned}$$

so that *q*(*t*) represents the earliest time at which $\chi (t)$ vaccines were administered by the ${\varvec{U}}$ policy. By continuity of the integral, this means that

$$\begin{aligned} \sum _{i=1}^nW_i(q(t)) = \int _0^{q(t)} \sum _{j=1}^n U_j(k)dk = \int _0^t\chi (s)ds. \end{aligned}$$

Define further

$$\begin{aligned} Q:= \sup \{t: q(t) < \infty \} \quad \text {and} \quad q_{\infty }:= \lim _{t \rightarrow Q}(q(t)) \end{aligned}$$

so that *Q* is the earliest time at which all of the vaccines given out by the ${\varvec{U}}$ policy could have been administered. Note that both *Q* and $q_{\infty }$ may be infinite. By taking the limit $t \rightarrow Q$, and noting the left-hand side is bounded by 1,

$$\begin{aligned} \int _0^{q_{\infty }}\sum _{j=1}^nU_j(k)dk= \int _0^Q\chi (k)dk \end{aligned}$$

Then, the integral of the new vaccination policy, $\tilde{{\varvec{W}}}$ is given by

$$\begin{aligned} {\tilde{W}}_i(t)= & {} \left\{ \begin{matrix} W_i(q(t)) &{} \text {if } t<Q\\ \\ W_i(q_{\infty }) + \frac{(N_i - W_i(q_{\infty }))\int _Q^t\chi (s)ds}{1-\sum _{i=1}^n W_i(q_{\infty })} &{} \text {if } \int _0^t \chi (s)ds < 1 \quad \text {and} \quad t \ge Q\\ \\ N_i &{}\text {if } \int _0^t \chi (s)ds \ge 1 \quad \text {and}\quad t \ge Q\\ \end{matrix}\right. . \end{aligned}$$

This is well-defined as

$$\begin{aligned} \sum _{i=1}^nW_i(q_{\infty }) = 1 \Rightarrow \int _0^Q\chi (s)ds = 1 \end{aligned}$$

and so, in this case, the second part of the definition of $\chi$ is never used. It is important to establish for feasibility that each $W_i$ is bounded by $N_i$.

**Claim:** ${\tilde{W}}_i(t) \le N_i$ for all $t \ge 0$ and all $i \in \{1,...,n\}$.

#### Proof

If $t < Q$, then $W_i(q(t)) \le N_i$ for all $t < Q$ by feasibility of ${\varvec{U}}$. Otherwise, if $t \ge Q$ and $\int _0^t\chi (s)ds < 1$, then one has

$$\begin{aligned} W_i(q_{\infty }) + \frac{(N_i - W_i(q_{\infty }))\int _Q^t\chi (s)ds}{1-\sum _{i=1}^n W_i(q_{\infty })}&\le W_i(q_{\infty }) + \frac{(N_i - W_i(q_{\infty }))(1 - \int _0^Q\chi (s)ds)}{1-\sum _{i=1}^n W_i(q_{\infty })} \\&= W_i(q_{\infty }) + \frac{(N_i - W_i(q_{\infty }))(1 - \sum _{i=1}^n W_i(q_{\infty }))}{1-\sum _{i=1}^n W_i(Q)}\\&= N_i \end{aligned}$$

while if $\int _0^t\chi (s)ds \ge 1$ then the result is immediate.

The optimisation problem is framed in terms of ${\varvec{U}}$ rather than ${\varvec{W}}$, and so it is important to show that there is some $\tilde{{\varvec{U}}}$ that integrates to $\tilde{{\varvec{W}}}$. One can do this by proving the Lipschitz continuity of ${\tilde{W}}_i$ for each *i*. $\square$

**Claim:** ${\tilde{W}}_i(t)$ is Lipschitz continuous for each $i \in \{1,...,n\}$

#### Proof

Note that for $s,t < Q$, if *M* is a bound for $\chi$ (which is assumed to exist)

$$\begin{aligned} \vert {\tilde{W}}_i(t) - {\tilde{W}}_i(s)\vert&= \bigg \vert \int _{q(s)}^{q(t)}U_i(k)dk\bigg \vert \\&\le \bigg \vert \int _{q(s)}^{q(t)}\sum _{j=1}^nU_j(k)dk\bigg \vert \\&= \bigg \vert \int _s^t\chi (k)dk\bigg \vert \\&\le \vert t-s\vert M \end{aligned}$$

Moreover, if $s,t > Q$ and $\int _0^t\chi (k)dk, \int _0^s\chi (k)dk < 1$, then

$$\begin{aligned} \vert {\tilde{W}}_i(t) - {\tilde{W}}_i(s)\vert \le \bigg \vert \frac{(N_i - W_i(q_{\infty }))\int _s^t\chi (s)ds}{1-\sum _{i=1}^n W_i(q_{\infty })}\bigg \vert \le M\bigg \vert \frac{(N_i - W_i(q_{\infty }))}{1-\sum _{i=1}^n W_i(q_{\infty })}\bigg \vert \vert t-s\vert \end{aligned}$$

and if $s,t > Q$ and $\int _0^t \chi (k)dk, \int _0^s\chi (k)dk \ge 1$, then ${\tilde{W}}_i(t) = {\tilde{W}}_i(s)$. The intermediate cases (where *s* and *t* correspond to different cases in the definition of $\chi$) can be proved by combining these bounds.

This means that (for each *i*) there exists a Lebesgue integrable function ${\tilde{U}}_i(t)$ such that

$$\begin{aligned} \frac{d{\tilde{W}}_i}{dt} = {\tilde{U}}_i(t) \quad \text {almost everywhere} \end{aligned}$$

and, for all $t \ge 0$

$$\begin{aligned} \int _0^t {\tilde{U}}_i(s)ds = {\tilde{W}}_i(t) \end{aligned}$$

A proof of this (for the broader class of absolutely continuous functions) can be found in Bárcenas (2000). One can set ${\tilde{U}}_i(t)$ to be zero for any *t* such that ${\tilde{W}}_i(t)$ is not differentiable. Thus, noting that, where it is differentiable, the derivative of ${\tilde{W}}_i$ is bounded by its Lipschitz constant, ${\tilde{U}}_i(t)$ is bounded as required.

Note that, in all cases (as $\sum _{i=1}^nN_i = 1$)

$$\begin{aligned} \sum _{i=1}^n{\tilde{W}}_i(t) = \min \bigg (\int _0^t\chi (s)ds, 1\bigg ) \end{aligned}$$

and so $\tilde{{\varvec{W}}}$ does correspond to a maximal vaccination rate. If $\chi (t)$ is continuous almost everywhere, then one can differentiate this relationship at *t* where each ${\tilde{W}}_i$ is differentiable and $\chi$ is continuous to show that $\sum _{i=1}^nU_i(t) = \chi (t)$. The complement of this set must have zero measure (as it is the finite union of zero measure sets), and so, in this case, one can change the values of each $U_i(t)$ so that $\sum _{i=1}^nU_i(t) = \chi (t)$ everywhere without changing the value of ${\varvec{W}}$. $\square$

**Claim:** ${\tilde{W}}_i(t) \ge W_i(t)$ for all $i \in \{1,...,n\}$ and $t \ge 0$

#### Proof

Note that, by maximality of $\chi$, for $t < Q$,

$$\begin{aligned} \sum _{j=1}^n{\tilde{W}}_i(t) = \sum _{j=1}^nW_j(q(t)) = \int _0^t\chi (s)ds \ge \sum _{j=1}^nW_j(t) \end{aligned}$$

If $q(t) \ge t$, then $W_i(q(t)) \ge W_i(t)$ for each *i*. If $q(t) < t$, then it is necessary that $W_i(q(t)) = W_i(t)$ for each *i* as $W_i$ is non-decreasing. Thus, $W_i(q(t)) \ge W_i(t)$ for all *i* and for all $t < Q$.

If $t > Q$ and $\int _0^t\chi (s)ds < 1$, then

25 $$\begin{aligned} {\tilde{W}}_i(t) \ge W_i(q_{\infty }) \end{aligned}$$

Now, by definition of *Q*, it is necessary that

$$\begin{aligned} \int _0^t \chi (k)dk \ge \int _0^{\infty }\sum _{j=1}^nU_j(k)dk \quad \forall t > Q \end{aligned}$$

as otherwise, there must exist some $t > Q$ and some $s < \infty$ such that

$$\begin{aligned} \int _0^t\chi (k)dk = \int _0^{s}\sum _{j=1}^nU_j(k)dk \end{aligned}$$

which means that $q(t) <\infty$. Thus, by continuity, for all $\tau \in (0,t)$, there exists some *s* such that

$$\begin{aligned} \int _0^{\tau }\chi (k)dk = \int _0^{s}\sum _{j=1}^nU_j(k)dk \end{aligned}$$

which means $Q \ge t$, which is a contradiction.

Thus, by taking $t \rightarrow Q$,

$$\begin{aligned} \int _0^{q_{\infty }} \sum _{i=1}^nU_i(k)dk = \int _0^Q\chi (k)dk \ge \int _0^{\infty }\sum _{j=1}^nU_j(k)dk \end{aligned}$$

and so

$$\begin{aligned} \int _{q_{\infty }}^{\infty }U_j(k)dk = 0 \Rightarrow W_i(t) = W_i(q_{\infty }) \quad \forall i\in \{1,...,n\} \quad \text {and} \quad \forall t \ge q_{\infty } \end{aligned}$$

Thus, using (25),

$$\begin{aligned} {\tilde{W}}_i(t) \ge W_i(t). \end{aligned}$$

Finally, if $t > Q$ and $\int _0^t\chi (s)ds \ge 1$, then ${\tilde{W}}_i(t) = N_i \ge W_i(t)$. Thus, for all *t* and *i*,

$$\begin{aligned} {\tilde{W}}_i(t) \ge W_i(t) \end{aligned}$$

as required.

Thus, by Theorem 1, it is necessary that

$$\begin{aligned} H({\varvec{U}}) \ge H(\tilde{{\varvec{U}}}) \end{aligned}$$

and hence, by the optimality of ${\varvec{U}}$, $\tilde{{\varvec{U}}}$ is optimal as required. $\square$

### Theorem 3

#### Theorem 3

Under the assumptions of Theorem 2, consider a modified objective function ${\mathcal {H}}$ given by

$$\begin{aligned} {\mathcal {H}}({\varvec{U}}) = H({\varvec{U}}) + F({\varvec{W}}(\infty )) \end{aligned}$$

for any function *F*. Then, with $\chi$ defined to be the maximal vaccination effort as in Theorem 2, there exists an optimal solution $\tilde{{\varvec{U}}}$ such that, for some $\tau \ge 0$

$$\begin{aligned} \sum _{i=1}^n{\tilde{W}}_i(t) =\left\{ \begin{matrix} \int _0^t \chi (s)ds&{} \text {if } t \le \tau \\ \\ W_i(\tau ) &{} \text {otherwise} \\ \end{matrix}\right. . \end{aligned}$$

Moreover, if $\chi$ is continuous almost everywhere, then there is an optimal solution $\tilde{{\varvec{U}}}$ such that

$$\begin{aligned} \sum _{i=1}^nU_i(t) =\left\{ \begin{matrix} \chi (t)&{} \text {if } t \le \tau \\ 0 &{} \text {otherwise} \\ \end{matrix}\right. . \end{aligned}$$

#### Proof

This follows directly from the proof of Theorem 2. One can again define $\tilde{{\varvec{U}}}$ in the interval (0, *Q*) (where *Q* is defined in the proof of Theorem 2) such that

$$\begin{aligned} H({\varvec{U}}) \ge H(\tilde{{\varvec{U}}}) \quad \text {and} \quad \int _0^t \sum _{i=1}^n{\tilde{U}}_i(s)ds = \int _0^t\chi (s)ds \quad \forall t < Q \end{aligned}$$

with the only difference being that now

$$\begin{aligned} {\tilde{U}}_i(t) = 0 \quad \forall t \ge Q. \end{aligned}$$

Thus, as shown in the proof of Theorem 2,

$$\begin{aligned} {\varvec{W}}(\infty ) = {\varvec{W}}(q_{\infty }) = \tilde{{\varvec{W}}}(Q) = \tilde{{\varvec{W}}}(\infty ) \end{aligned}$$

and so

$$\begin{aligned} {\mathcal {H}}({\varvec{U}}) \ge {\mathcal {H}}(\tilde{{\varvec{U}}}), \end{aligned}$$

which means $\tilde{{\varvec{U}}}$ is optimal as required. $\square$

## Supplementary Lemmas For Propositions A.1.1 and A.2.1 and Theorem 2

For the proofs of these lemmas, it is helpful to recall the following definitions of the following variables, which will be extensively used.

$$\begin{aligned} K_{ij}(t)= & {} \frac{\beta ^1_{ij}}{\mu ^1_j}R_j + \frac{\beta ^2_{ij}}{\mu ^2_j}R^V_j, \\ L_{ij}(t):= & {} \frac{\beta _{ij}^3}{\mu _i^1} R_j + \frac{\beta _{ij}^4}{\mu _i^2}R^V_j \end{aligned}$$

and

$$\begin{aligned} \Pi := \left\{ i: \exists t\ge 0 \quad \text {s.t.} \quad I_i(t)> 0 \quad \text {or} \quad I_i^V(t) > 0\right\} . \end{aligned}$$

Moreover, note that, under the assumptions of Proposition A.1.1 and A.2.1, each $U_i(t)$ is a step function and is therefore piecewise smooth in each bounded interval. Thus, in particular, the derivatives of each of the model variables (and indeed, the derivative of $W_i(t)$) are piecewise continuous in each bounded interval, meaning that each of the model variables is piecewise continuously differentiable in each bounded interval. This means that integration by parts can be performed (in a bounded interval), as will be done extensively throughout the proofs of these lemmas.

### Lemma B.1

#### Lemma B.1

Suppose that *f*(*t*) is a non-increasing, non-negative, continuous and piecewise continuously differentiable function and that the continuous and piecewise continuously differentiable functions *g*(*t*) and *h*(*t*) satisfy $g(0) = h(0)$ and $g(t) \le h(t)$ for all $t\ge 0$. Then,

$$\begin{aligned} \int _0^t g'(s)f(s) ds \le \int _0^t h'(s) f(s) ds. \end{aligned}$$

#### Proof

This follows from integrating by parts:

$$\begin{aligned} \int _0^t g'(s)f(s) ds&= g(t)f(t) - g(0)f(0) - \int _0^t g(s)f'(s) ds \\&= g(t)f(t) - h(0)f(0) + \int _0^t g(s)\vert f'(s)\vert ds \\&\le h(t)f(t) - h(0)f(0) + \int _0^t h(s)\vert f'(s)\vert ds \\&\le h(t)f(t) - h(0)f(0) - \int _0^t h(s)f'(s) ds \\&= \int _0^t h'(s)f(s) ds \end{aligned}$$

as required. $$\square $$

### Lemma B.2

#### Lemma B.2

Suppose that

$$\begin{aligned}{} & {} \sum _{j=1}^n K_{ij}(t) \ge \sum _{j=1}^n {\tilde{K}}_{ij}(t) \quad \text {and} \quad \sum _{j=1}^n L_{ij}(t)\\ {}{} & {} \ge \sum _{j=1}^n {\tilde{L}}_{ij}(t) \quad \forall i \in \{1,...,n\} \quad \text {and} \quad t \in [0,T]. \end{aligned}$$

Then,

$$\begin{aligned} S_i(t) + S_i^V(t) \le {\tilde{S}}_i(t) + {\tilde{S}}_i^V(t) \quad \forall t \in [0,T]. \end{aligned}$$

#### Proof

To reduce notation in this proof, define

$$\begin{aligned} {\mathcal {K}}(t):= \sum _{j=1}^nK_{ij}(t) \quad \text {and} \quad {\mathcal {L}}(t):= \sum _{j=1}^nL_{ij}(t) \end{aligned}$$

Note that

$$\begin{aligned} \frac{d}{dt}(S_i+S^V_i)&= -\sum _{j=1}^n\bigg (\beta ^1_{ij} I_j + \beta _{ij}^2I^V_j\bigg )S_i - \sum _{j=1}^n\bigg (\beta _{ij}^3I_j + \beta _{ij}^4I_j^V\bigg )S_i^V\\&= -\sum _{j=1}^n\bigg (\beta _{ij}^3 I_j + \beta _{ij}^4I^V_j\bigg )(S_i + S_i^V)...\\&\quad -\sum _{j=1}^n \bigg ((\beta ^1_{ij} - \beta _{ij}^3)I_j + (\beta _{ij}^2 - \beta _{ij}^4)I_j^V\bigg )S_i. \end{aligned}$$

Thus,

$$\begin{aligned} -\sum _{j=1}^n \bigg ((\beta ^1_{ij} - \beta _{ij}^3)I_j + (\beta _{ij}^2 - \beta _{ij}^4)I_j^V\bigg )S_i&= \frac{d}{dt}(S_i+S^V_i)...\\&\quad +\sum _{j=1}^n\bigg (\beta _{ij}^3 I_j + \beta _{ij}^4I^V_j\bigg )(S_i + S_i^V)\\&= \frac{d}{dt}\bigg ((S_i + S_i^V)e^{{\mathcal {L}}(t)}\bigg ) e^{-{\mathcal {L}}(t)}. \end{aligned}$$

This means that

$$\begin{aligned} S_i(t) + S_i^V(t)&= e^{-{\mathcal {L}}(t)}\left[ S_i(0) - \int _0^te^{{\mathcal {L}}(s)} \sum _{j=1}^n \bigg ((\beta ^1_{ij} - \beta _{ij}^3)I_j + (\beta _{ij}^2 - \beta _{ij}^4)I_j^V\bigg )S_ids\right] \\ \\&=S_i(0)\left[ e^{-{\mathcal {L}}(t)} - \int _0^te^{{\mathcal {L}}(s) - {\mathcal {K}}(s) - {\mathcal {L}}(t)} ({\mathcal {K}}'(s) - {\mathcal {L}}'(s) )\left( \frac{N_i-W_i(s)}{N_i}\right) \right] ds. \end{aligned}$$

Now, one can see that, as $0\le W_i(s) \le N_i$,

$$\begin{aligned} 0\le \frac{N_i - W_i(s)}{N_i} \le 1 \quad \forall s \ge 0 \end{aligned}$$

and hence

$$\begin{aligned} e^{-{\mathcal {L}}(t)} = 1 - \int _0^t {\mathcal {L}}'(s)e^{-{\mathcal {L}}(s)}ds \le 1 - \int _0^t{\mathcal {L}}'(s)e^{-{\mathcal {L}}(s)}\left( \frac{N_i-W_i(s)}{N_i}\right) ds. \end{aligned}$$

Now, this means that

$$\begin{aligned}&S_i(t) + S_i^V(t) \\&\quad \le S_i(0) - S_i(0)\int _0^t\bigg [{\mathcal {L}}'(s)e^{-{\mathcal {L}}(s)}\\&\qquad + e^{{\mathcal {L}}(s) - {\mathcal {K}}(s) - {\mathcal {L}}(t)} \sum _{j=1}^n\bigg ( {\mathcal {K}}'(s) - {\mathcal {L}}'(s)\bigg ) \bigg ]\left( \frac{N_i-W_i(s)}{N_i}\right) ds. \end{aligned}$$

This allows the use of Lemma B.1. Firstly, note that, as ${\mathcal {K}}'(s) \ge {\mathcal {L}}'(s) \ge 0$ and ${\tilde{W}}_i(s) \ge W_i(s)$, one has

$$\begin{aligned}&S_i(t) + S_i^V(t) \\&\quad \le S_i(0) - S_i(0)\int _0^t\bigg [{\mathcal {L}}'(s)e^{-{\mathcal {L}}(s)}\\&\qquad + e^{{\mathcal {L}}(s) - {\mathcal {K}}(s) - {\mathcal {L}}(t)} \sum _{j=1}^n\bigg ( {\mathcal {K}}'(s) - {\mathcal {L}}'(s)\bigg ) \bigg ]\left( \frac{N_i-{\tilde{W}}_i(s)}{N_i}\right) ds. \end{aligned}$$

Moreover,

$$\begin{aligned}&\int _0^t\bigg [{\mathcal {L}}'(s)e^{-{\mathcal {L}}(s)} + e^{{\mathcal {L}}(s) - {\mathcal {K}}(s) - {\mathcal {L}}(t)} \sum _{j=1}^n\bigg ( {\mathcal {K}}'(s) - {\mathcal {L}}'(s)\bigg ) \bigg ]ds \\&\quad = 1 - e^{-{\mathcal {L}}(t)} + e^{-{\mathcal {L}}(t)} - e^{-{\mathcal {K}}(t)}\\&\quad = 1 -e^{-{\mathcal {K}}(t)}\\&\quad \ge 1 -e^{-\tilde{{\mathcal {K}}}(t)}\\&\quad \ge \int _0^t\bigg [\tilde{{\mathcal {L}}}'(s)e^{-\tilde{{\mathcal {L}}}(s)} + e^{\tilde{{\mathcal {L}}}(s) - \tilde{{\mathcal {K}}}(s) - \tilde{{\mathcal {L}}}(t)} \sum _{j=1}^n\bigg ( \tilde{{\mathcal {K}}}'(s) - \tilde{{\mathcal {L}}}'(s)\bigg ) \bigg ]ds \\ \end{aligned}$$

and $N_i - W_i(s)$ is non-increasing in *s*. Thus, by Lemma B.1, with

$$\begin{aligned} g(s) = 1 - e^{-{\mathcal {L}}(s)} +e^{-{\mathcal {L}}(t)} - e^{{\mathcal {L}}(s) -{\mathcal {K}}(s)-{\mathcal {L}}(t)}, \end{aligned}$$

*h*(*s*) defined as the tilde version of *g*(*s*), and $f(s):= N_i - W_i(s)$, one has

26 $$\begin{aligned}&\int _0^t\bigg [{\mathcal {L}}'(s)e^{-{\mathcal {L}}(s)} + e^{{\mathcal {L}}(s) - {\mathcal {K}}(s) - {\mathcal {L}}(t)} \sum _{j=1}^n\bigg ( {\mathcal {K}}'(s) - {\mathcal {L}}'(s)\bigg ) \bigg ]\left( \frac{N_i-{\tilde{W}}_i(s)}{N_i}\right) ds \nonumber \\&\quad \ge \int _0^t\bigg [\tilde{{\mathcal {L}}}'(s)e^{-\tilde{{\mathcal {L}}}(s)} + e^{\tilde{{\mathcal {L}}}(s) - \tilde{{\mathcal {K}}}(s) - \tilde{{\mathcal {L}}}(t)} \sum _{j=1}^n\bigg ( \tilde{{\mathcal {K}}}'(s) - \tilde{{\mathcal {L}}}'(s)\bigg ) \bigg ]\left( \frac{N_i-{\tilde{W}}_i(s)}{N_i}\right) ds . \end{aligned}$$

Thus, (as this integral is multiplied by -1 in (26)), combining this with (26) gives

$$\begin{aligned} S_i(t) + S_i^V(t) \le {\tilde{S}}_i(t) + {\tilde{S}}_i^V(t) \quad \forall t \in [0,T] \end{aligned}$$

as required $$\square $$

### Lemma B.3

#### Lemma B.3

Suppose that

$$\begin{aligned} \sum _{j=1}^n K_{ij}(t) \ge \sum _{j=1}^n {\tilde{K}}_{ij}(t) \quad \forall i \in \{1,...,n\} \quad \text {and} \quad t \in [0,T]. \end{aligned}$$

Then

$$\begin{aligned} I_i(t) + R_i(t) \ge {\tilde{I}}_i(t) + {\tilde{R}}_i(t) \quad \forall t \in [0,T]. \end{aligned}$$

To begin, one can write the equation for $S_i$ as

$$\begin{aligned} \frac{1}{S_i}\frac{dS_i}{dt} = -\sum _{j=1}^n(K'_{ij}(t)) - \frac{U_i}{N_i - W_i} \end{aligned}$$

and hence, integrating

$$\begin{aligned} \ln (S_i(t)) - \ln (S_i(0)) = -\sum _{j=1}^nK_{ij}(t) + \ln (N_i - W_i(t)) - \ln (N_i) \end{aligned}$$

which implies

$$\begin{aligned} S_i(t) = \bigg (\frac{S_i(0)(N_i - W_i(t))}{N_i}\bigg )e^{-\sum _{j=1}^nK_{ij}(t)} \end{aligned}$$

Using this result shows that

$$\begin{aligned} \frac{d}{dt}(I_i + R_i)&= \sum _{j=1}^n \bigg (\beta ^1_{ij}I_j + \beta _{ij}^2I^V_j\bigg ) S_i \\&= \sum _{j=1}^n K'_{ij}(t)S_i \\&=\bigg [ \sum _{j=1}^n K'_{ij}(t)\bigg ]\left( \frac{S_i(0)(N_i-W_i(t))}{N_i}\right) e^{-\sum _{j=1}^nK_{ij}(t)}, \end{aligned}$$

Thus,

27 $$\begin{aligned} I_i(t) + R_i(t)&= I_i(0) + \int _0^t\bigg [ \sum _{j=1}^n K'_{ij}(s)\bigg ]\left( \frac{S_i(0)(N_i-W_i(s))}{N_i}\right) e^{-\sum _{j=1}^nK_{ij}(s)}ds\nonumber \\&\ge {\tilde{I}}_i(0) + \int _0^t\bigg [ \sum _{j=1}^n K'_{ij}(s)\bigg ]\left( \frac{S_i(0)(N_i-{\tilde{W}}_i(s))}{N_i}\right) e^{-\sum _{j=1}^nK_{ij}(s)}ds, \end{aligned}$$

using the fact that the initial conditions are the same in both cases and that $W_i \le {\tilde{W}}_i$. Now, one can use the results of Lemma B.1 with

$$\begin{aligned} g(t) = 1-\exp \bigg (-\sum _{j=1}^nK_{ij}(t)\bigg ),\quad h(t) =1-\exp \bigg (-\sum _{j=1}^n{\tilde{K}}_{ij}(t)\bigg ) \end{aligned}$$

and $f(t) = (N_i - {\tilde{W}}_i(t))$, noting that

$$\begin{aligned} \int _0^t\bigg [ \sum _{j=1}^n K'_{ij}(s)\bigg ]e^{-\sum _{j=1}^nK_{ij}(s)}ds&= 1-e^{-\sum _{j=1}^nK_{ij}(t)} \\&\ge 1-e^{-\sum _{j=1}^n{\tilde{K}}_{ij}(t)}\\&=\int _0^t\bigg [ \sum _{j=1}^n {\tilde{K}}'_{ij}(s)\bigg ]e^{-\sum _{j=1}^n{\tilde{K}}_{ij}(s)}ds \end{aligned}$$

and that $N_i-{\tilde{W}}_i(t)$ is non-increasing. Thus,

$$\begin{aligned} I_i(t) + R_i(t) \ge {\tilde{I}}_i(t) + {\tilde{R}}_i(t) \quad \forall t \in [0,T] \end{aligned}$$

as required.

### Lemma B.4

#### Lemma B.4

Suppose that

$$\begin{aligned} \sum _{j=1}^n K_{ij}(t) \ge \sum _{j=1}^n {\tilde{K}}_{ij}(t) \quad \forall i \in \{1,...,n\} \quad \text {and} \quad t \in [0,T]. \end{aligned}$$

Then,

$$\begin{aligned} R_i(t) \ge {\tilde{R}}_i(t) \quad \forall t \in [0,T] \end{aligned}$$

#### Proof

The result of Lemma B.3 can be written as

$$\begin{aligned} \frac{1}{\mu ^1_i}\frac{dR_i}{dt} + R_i\ge \frac{1}{\mu ^1_i}\frac{d{\tilde{R}}_i}{dt} + {\tilde{R}}_i \quad \forall t \in [0,T] \end{aligned}$$

which implies

$$\begin{aligned} \frac{d}{dt}\left( R_ie^{\mu ^1_i t}\right) \ge \frac{d}{dt}\left( {\tilde{R}}_ie^{\mu ^1_i t}\right) \end{aligned}$$

and hence, after integrating and cancelling exponentials, one finds

$$\begin{aligned} R_i(t) \ge {\tilde{R}}_i(t) \quad \forall t \in [0,T] \end{aligned}$$

as required. $$\square $$

### Lemma B.5

#### Lemma B.5

Suppose that

$$\begin{aligned} T:= \inf \left\{ t: K_{ij}(t)<{\tilde{K}}_{ij}(t) \quad \text {or}\quad L_{ij}(t) < {\tilde{L}}_{ij}(t) \quad \text {for some } i,j \in \{1,...,n\} \right\} \end{aligned}$$

exists. Then, for some $b \in \{1,...,n\}$, and some real constants $\kappa$ and $\eta$,

$$\begin{aligned} R_b(T) + \kappa R^V_b(T)= & {} {\tilde{R}}_b(T) + \kappa {\tilde{R}}^V_b(T), \\ I_b(T) +\eta I^V_b(T)\le & {} {\tilde{I}}_b(T) + \eta {\tilde{I}}^V_b(T) \end{aligned}$$

and

$$\begin{aligned} 0 \le \kappa \le \eta \le 1 \end{aligned}$$

#### Proof

Suppose that *T* exists. Then, by continuity, there exists some *a* and *b* such that $K_{ab}(T) = {\tilde{K}}_{ab}(T)$ or $L_{ab}(T) = {\tilde{L}}_{ab}(T)$. These can be rearranged to give, respectively,

$$\begin{aligned} R_b(T) + \frac{\mu _b^1\beta ^2_{ab}}{\mu ^2_b\beta _{ab}^1}R^V_b(T) = {\tilde{R}}_b(T) + \frac{\mu _b^1\beta ^2_{ab}}{\mu ^2_b\beta _{ab}^1} {\tilde{R}}^V_b(T) \end{aligned}$$

or

$$\begin{aligned} R_b(T) + \frac{\mu _b^1\beta ^4_{ab}}{\mu ^2_b\beta _{ab}^3}R^V_b(T) = {\tilde{R}}_b(T) + \frac{\mu _b^1\beta ^4_{ab}}{\mu ^2_b\beta _{ab}^3} {\tilde{R}}^V_b(T). \end{aligned}$$

This can be written as

$$\begin{aligned} R_b(T) + \kappa R^V_b(T) = {\tilde{R}}_b(T) + \kappa {\tilde{R}}^V_b(T), \end{aligned}$$

where, by the inequality constraints on the $\beta _{ij}^{\alpha }$ and $\mu _i^{\alpha }$

28 $$\begin{aligned} \kappa \le \frac{\mu _b^1}{\mu _b^2}. \end{aligned}$$

Moreover, note that

$$\begin{aligned} \frac{d}{dt}\left( R_b + \kappa R^V_b \right) = \mu _b^1 I_b + \frac{\beta ^2_{ab}\mu _b^1}{\beta _{ab}^1}I^V_b \end{aligned}$$

is a continuous function. Thus, if

$$\begin{aligned} \frac{d}{dt}\left( R_b + \kappa R^V_b \right) \bigg \vert _{t=T} > \frac{d}{dt}\left( {\tilde{R}}_b + \kappa {\tilde{R}}^V_b \right) \bigg \vert _{t=T}, \end{aligned}$$

then there exists some $\tau > 0$ such that

$$\begin{aligned} \int _T^{T+\tau } \frac{d}{dt}\bigg ( R_b(s) + \kappa R^V_b(s) \bigg )ds > \int _T^{T+\tau }\frac{d}{dt}\bigg ( {\tilde{R}}_b(s) +\kappa {\tilde{R}}^V_b(s) \bigg )ds \quad \forall t\in [0,\tau ] \end{aligned}$$

and hence, in particular

$$\begin{aligned} R_b(T+t) + \kappa R^V_b(T+t) > {\tilde{R}}_b(T+t) + \kappa {\tilde{R}}^V_b(T+t) \quad \forall t \in [0,\tau ], \end{aligned}$$

Thus, it is necessary that there is some *b* such that

$$\begin{aligned} \frac{d}{dt}\left( R_b + \kappa R^V_b \right) \bigg \vert _{t=T}\le \frac{d}{dt}\left( {\tilde{R}}_b + \kappa {\tilde{R}}^V_b \right) \bigg \vert _{t=T} \end{aligned}$$

so

$$\begin{aligned} I_b(T) + \frac{\kappa \mu _b^2}{\mu _b^1}I^V(T) \le {\tilde{I}}_b(T) + \frac{\kappa \mu _b^2}{\mu _b^1} {\tilde{I}}^V_b(T). \end{aligned}$$

This can be written as

$$\begin{aligned} I_b(t) +\eta I^V_b(t) \le {\tilde{I}}_b(t) + \eta {\tilde{I}}^V_b(t), \end{aligned}$$

where, by (28), the fact that $\mu _b^2 \ge \mu _b^1$, and the non-negativity of all parameters,

$$\begin{aligned} 0\le \kappa \le \eta \le 1. \end{aligned}$$

as required $$\square $$

### Lemma B.6

For the purposes of this lemma, it is helpful to recall the inequality system (9)–(15).

7 $$\begin{aligned} S_b(T)+S^V_b(T)&\le {\tilde{S}}_b(T) + {\tilde{S}}^V_b(T), \end{aligned}$$

8 $$\begin{aligned} I_b(T) + R_b(T)&\ge {\tilde{I}}_b(T) + {\tilde{R}}_b(T) \end{aligned}$$

9 $$\begin{aligned} R_b(T)&\ge {\tilde{R}}_b(T), \end{aligned}$$

10 $$\begin{aligned} R_b(T) + \kappa R^V_b(T)&\le {\tilde{R}}_b(T) + \kappa {\tilde{R}}^V_b(T), \end{aligned}$$

11 $$\begin{aligned} I_b(T) + \eta I^V_b(T)&\le {\tilde{I}}_b(T) + \eta {\tilde{I}}^V_b(T), \end{aligned}$$

12 $$\begin{aligned} 0 \le \kappa&\le \eta \le 1. \end{aligned}$$

and

13 $$\begin{aligned}&S_b(T) + I_b(T) + R_b(T) + S^V_b(T) + I^V_b(T) + R^V_b(T) \\&\quad = {\tilde{S}}_b(T) + {\tilde{I}}_b(T) + {\tilde{R}}_b(T) + {\tilde{S}}^V_b(T)+ {\tilde{I}}^V_b(T) + {\tilde{R}}^V_b(T), \end{aligned}$$

#### Lemma B.6

Suppose that the system (9) - (15) holds for some $b \in \{1,...,n\}$ and some $T \ge 0$. Then,

$$\begin{aligned} \eta I^V_b(T) + \kappa R^V_b(T)&= \eta {\tilde{I}}^V_b(T) + \kappa {\tilde{R}}^V_b(T)\\ I_b(T) + R_b(T)&= {\tilde{I}}_b(T) + {\tilde{R}}_b(T)\\ I_b^V(T) + R_b^V(T)&= {\tilde{I}}^V_b(T) + {\tilde{R}}^V_b(T)\\ S_b(T) + S_b^V(T)&= {\tilde{S}}_b(T) + {\tilde{S}}_b^V(T). \end{aligned}$$

#### Proof

To begin, note that adding inequalities (9), (12) and (13) gives

$$\begin{aligned}&S_b(T)+S^V_b(T) + R_b(T) + \kappa R^V_b(T) + I_b(T) + \eta I^V_b(T) \\&\quad \le {\tilde{S}}_b(T) + {\tilde{S}}^V_b(T) +{\tilde{R}}_b(T) + \kappa {\tilde{R}}^V_b(T)+{\tilde{I}}_b(T) + \eta {\tilde{I}}^V_b(T) \end{aligned}$$

and then, using (15) shows that

29 $$\begin{aligned} (\kappa -1)R^V_b(T) + (\eta -1)I^V_b(T) \le (\kappa -1){\tilde{R}}^V_b(T) + (\eta -1){\tilde{I}}^V_b(T). \end{aligned}$$

Moreover, adding (12) and (13) shows that

$$\begin{aligned} I_b(T) + \eta I^V_b(T) + R_b(T) + \kappa R^V_b(T) \le {\tilde{I}}_b(T) + \eta {\tilde{I}}^V_b(T) + {\tilde{R}}_b(T) + \kappa {\tilde{R}}^V_b(T) \end{aligned}$$

and then, using (10) shows that

30 $$\begin{aligned} \eta I^V_b(T) + \kappa R^V_b(T) \le \eta {\tilde{I}}^V_b(T) + \kappa {\tilde{R}}^V_b(T). \end{aligned}$$

Now, from the inequality (12) combined with the inequality (11), it must be the case that

31 $$\begin{aligned} R^V_b(T) - {\tilde{R}}^V_b(T) \le \frac{1}{\kappa }({\tilde{R}}_b(T) - R_b(T)) \le 0. \end{aligned}$$

Define

$$\begin{aligned} x: = R^V_b(T) - {\tilde{R}}^V_b(T) \quad \text {and} \quad y:= I^V_b(T) - {\tilde{I}}^V_b(T) \end{aligned}$$

so that the system given by (14), (29), (30) and (31) reduces to

32 $$\begin{aligned} (\kappa - 1)x + (\eta -1)y&\le 0 \end{aligned}$$

33 $$\begin{aligned} \kappa x + \eta y&\le 0 \nonumber \\ x&\le 0 \nonumber \\ 0\le \kappa \le \eta&\le 1. \end{aligned}$$

Note first that $x = 0$ implies that $y = 0$ as $\eta$ and $(\eta -1)$ have different signs. Thus, in this case, the inequalities (32) and (33) are in fact equalities.

Suppose instead that $x \ne 0$ (so $x < 0$). The first two of these inequalities can be rearranged (noting the signs of the denominators) to give

$$\begin{aligned} -\frac{(\kappa - 1)x}{(\eta - 1)} \le y \le \frac{-\kappa x}{\eta } \end{aligned}$$

and so, as $-x > 0$,

34 $$\begin{aligned} \frac{(\kappa - 1)}{(\eta - 1)} \le -\frac{y}{x} \le \frac{\kappa }{\eta }. \end{aligned}$$

However, note that

$$\begin{aligned} \kappa< \eta&\Rightarrow \eta \kappa - \eta< \eta \kappa - \kappa \\&\Rightarrow \eta (\kappa - 1) < \kappa (\eta - 1)\\&\Rightarrow \frac{\kappa - 1}{\eta - 1} > \frac{\kappa }{\eta } \end{aligned}$$

and hence, as $\kappa \le \eta$, for there to be solutions to the inequality (34), it is necessary that

$$\begin{aligned} \kappa = \eta \Rightarrow \frac{-y}{x} = 1 \Rightarrow y = -x. \end{aligned}$$

This means that the inequalities (32) and (33) are satisfied to equality in this and hence, from before, all cases. Thus, it is necessary that

35 $$\begin{aligned} (\kappa -1)R^V_b(T) + (\eta -1)I^V_b(T) = (\kappa -1){\tilde{R}}^V_b(T) + (\eta -1){\tilde{I}}^V_b(T) \end{aligned}$$

and

36 $$\begin{aligned} \eta I^V_b(T) + \kappa R^V_b(T) = \eta {\tilde{I}}^V_b(T) + \kappa {\tilde{R}}^V_b(T), \end{aligned}$$

which is the first required equality. Thus, one can once again add the inequalities (12) and (13) to give

$$\begin{aligned} I_b(T)+ R_b(T) + \bigg [\eta I^V_b(T) + \kappa R^V_b(T)\bigg ] \le {\tilde{I}}_b(T) +{\tilde{R}}_b(T) + \bigg [ \eta {\tilde{I}}^V_b(T) + \kappa {\tilde{R}}^V_b(T)\bigg ] \end{aligned}$$

and so

37 $$\begin{aligned} I_b(T)+ R_b(T) \le {\tilde{I}}_b(T) +{\tilde{R}}_b(T), \end{aligned}$$

which, combined with (10), shows that

38 $$\begin{aligned} I_b(T) + R_b(T) = {\tilde{I}}_b(T) + {\tilde{R}}_b(T). \end{aligned}$$

Moreover, one can subtract (35) from (36) to get

$$\begin{aligned} I_b^V(T) + R_b^V(T) = {\tilde{I}}^V_b(T) + {\tilde{R}}^V_b(T) \end{aligned}$$

and then, using (15) alongside (37) and (38) shows

$$\begin{aligned} S_b(T) + S_b^V(T) = {\tilde{S}}_b(T) + {\tilde{S}}_b^V(T) \end{aligned}$$

as required. $$\square $$

### Lemma B.7

Note that for this lemma, it will be assumed that each $K_{ij}(t) \ge {\tilde{K}}_{ij}(t)$, rather than the inequality simply holding for their sums as before.

#### Lemma B.7

Under the assumptions of Proposition A.1.1, suppose that the system of inequalities (9)–(15) holds for some $b \in \{1,...,n\}$ and some $T > 0$. Suppose further that

$$\begin{aligned} K_{ij}(t) \ge {\tilde{K}}_{ij}(t) \quad \forall i,j \in \{1,...,n\}. \end{aligned}$$

Then,

$$\begin{aligned} W_i(t) = {\tilde{W}}_i(t) \quad \forall i \in \{1,...,n\} \quad \text {and} \quad \forall t \in [0,T]. \end{aligned}$$

#### Proof

By Lemma B.6, the system (16)–(18) must hold for *b*. Now, Equation (27) in the proof of Lemma B.3 shows that

39 $$\begin{aligned} I_b(T) + R_b(T)&=\frac{S_b(0)}{N_b}\int _0^T\bigg [ \sum _{k=1}^n K'_{bk}(s)\bigg ] (N_b - W_b(s))e^{-\sum _{j=1}^nK_{bk}(s)}ds. \end{aligned}$$

Now, the equality (16) shows

$$\begin{aligned} I_b(T) + R_b(T) = {\tilde{I}}_b(T) + {\tilde{R}}_b(T) \end{aligned}$$

and hence, after cancelling the non-zero $S_b(0)$ and $N_b$ terms, (39) (and its tilde equivalent) shows that

40 $$\begin{aligned}&\int _0^T\bigg [ \sum _{k=1}^n K'_{bk}(s)\bigg ] (N_b - W_b(s))e^{-\sum _{k=1}^nK_{bk}(s)}ds \nonumber \\&\quad = \int _0^T\bigg [ \sum _{k=1}^n {\tilde{K}}'_{bk}(s)\bigg ](N_b - {\tilde{W}}_b(s))e^{-\sum _{k=1}^n{\tilde{K}}_{bk}(s)}ds. \end{aligned}$$

Note that, from Lemma C.6, as $\Pi = \{1,...,n\}$

$$\begin{aligned} {\tilde{I}}_k(s), I_k(s)> 0 \quad \forall k \in \{1,...,n\} \quad \text {and} \quad s > 0. \end{aligned}$$

Thus,

$$\begin{aligned} K'_{bk}(t) \ge \beta _{bk}^1I_j(t)> 0 \quad \forall t > 0. \end{aligned}$$

In particular,

$$\begin{aligned} \bigg [ \sum _{j=1}^n K'_{bk}(s)\bigg ] e^{-\sum _{j=1}^nK_{bk}(s)} > 0 \quad \forall s \in [0,T]. \end{aligned}$$

Moreover, by continuity of $K'_{ik}$ (as continuous functions attain their bounds on closed intervals), there exists some $m > 0$ such that

$$\begin{aligned} \bigg [ \sum _{k=1}^n K'_{bk}(s)\bigg ] e^{-\sum _{k=1}^nK_{bk}(s)} > m \quad \forall s \in [0,T]. \end{aligned}$$

Hence, as $$W_b \le {\tilde{W}}_b$$

41 $$\begin{aligned}&\int _0^T\bigg [ \sum _{k=1}^n K'_{bk}(s)\bigg ] (N_b - W_b(s))e^{-\sum _{k=1}^nK_{bk}(s)}ds \nonumber \\&\quad = \int _0^T\bigg [ \sum _{k=1}^n K'_{bk}(s)\bigg ] (N_b - {\tilde{W}}_b(s) + ({\tilde{W}}_b(s) - W_b(s))e^{-\sum _{k=1}^nK_{bk}(s)}ds\nonumber \\&\quad \ge \int _0^T\bigg [ \sum _{k=1}^n K'_{bk}(s)\bigg ] (N_b - {\tilde{W}}_b(s) )e^{-\sum _{k=1}^nK_{bk}(s)}ds + m\int _0^T{\tilde{W}}_b(s) - W_b(s)ds. \end{aligned}$$

Finally, as $N - {\tilde{W}}_b$ is decreasing and for any $t \in [0,T]$,

$$\begin{aligned} \int _0^t \bigg [ \sum _{k=1}^n K'_{bk}(s)\bigg ]e^{-\sum _{k=1}^nK_{bk}(s)}ds \ge \int _0^t \bigg [ \sum _{k=1}^n {\tilde{K}}'_{bk}(s)\bigg ]e^{-\sum _{k=1}^n{\tilde{K}}_{bk}(s)}ds \end{aligned}$$

one has, by Lemma B.1, setting

$$\begin{aligned} g(t)= & {} 1 - e^{-\sum _{k=1}^nK_{bk}(t)}, \quad h(t) = e^{-\sum _{k=1}^n{\tilde{K}}_{bk}(s)} \end{aligned}$$

and $$f(t) = N_b - {\tilde{W}}_b(s)$$,

$$\begin{aligned}&\int _0^T\bigg [ \sum _{k=1}^n K'_{bk}(s)\bigg ] (N_b - {\tilde{W}}_b(s) )e^{-\sum _{k=1}^nK_{bk}(s)}ds \\&\quad \ge \int _0^T\bigg [ \sum _{k=1}^n {\tilde{K}}'_{bk}(s)\bigg ] (N_b - {\tilde{W}}_b(s) )e^{-\sum _{k=1}^n{\tilde{K}}_{bk}(s)}ds \\&\quad = {\tilde{I}}_b(T) + {\tilde{R}}_b(T) \end{aligned}$$

and so, combining this with (41),

$$\begin{aligned} I_b(T) + R_b(T)\ge & {} {\tilde{I}}_b(T) + {\tilde{R}}_b(T) + m\int _0^T{\tilde{W}}_b(s) - W_b(s)ds \\\ge & {} {\tilde{I}}_b(T) + {\tilde{R}}_b(T) = I_b(T) + R_b(T). \end{aligned}$$

Hence,

$$\begin{aligned} \int _0^T{\tilde{W}}_b(s) - W_b(s)ds = 0, \end{aligned}$$

which by continuity means

$$\begin{aligned} W_b(t) = {\tilde{W}}_b(t) \quad \forall t \in [0,T] \end{aligned}$$

Now, moreover, substituting this back into the equality given in (40) shows that

$$\begin{aligned}&\int _0^T\bigg [ \sum _{k=1}^n K'_{bk}(s)\bigg ] (N_b - W_b(s))e^{-\sum _{k=1}^nK_{bk}(s)}ds \\&\quad = \int _0^T\bigg [ \sum _{k=1}^n {\tilde{K}}'_{bk}(s)\bigg ](N_b - W_b(s))e^{-\sum _{k=1}^n{\tilde{K}}_{bk}(s)}ds. \end{aligned}$$

Hence, integrating by parts, this shows that

$$\begin{aligned} 0&= (N_b - W_b(T))(e^{-\sum _{k=1}^nK_{bk}(T)} - e^{-\sum _{k=1}^n{\tilde{K}}_{bk}(T)})...\\&\quad +\int _0^TU_b(s)(e^{-\sum _{k=1}^nK_{bk}(s)} - e^{-\sum _{k=1}^n{\tilde{K}}_{bk}(s)})ds \end{aligned}$$

Now,

$$\begin{aligned} \sum _{k=1}^n{\tilde{K}}_{bk}(s)\ge \sum _{k=1}^nK_{bk}(s) \quad \forall s \in [0,T] \end{aligned}$$

and so, for equality, it is necessary that

$$\begin{aligned} (N_b - W_b(T))(e^{-\sum _{k=1}^nK_{bk}(T)} - e^{-\sum _{k=1}^n{\tilde{K}}_{bk}(T)}) = 0 \end{aligned}$$

Thus, as it is assumed that $W_b(t) < N_b$ for all $t\ge 0$,

$$\begin{aligned} e^{-\sum _{k=1}^nK_{bk}(T)} - e^{-\sum _{k=1}^n{\tilde{K}}_{bk}(T)} = 0 \end{aligned}$$

and hence, as $K_{bk}(T) \ge {\tilde{K}}_{bk}(T)$ for all $k \in \{1,...,n\}$,

42 $$\begin{aligned} K_{bk}(T) = {\tilde{K}}_{bk}(T) \quad \forall k \in \{1,...,n\} \end{aligned}$$

Now, suppose that $K'_{bk}(T) > {\tilde{K}}'_{bk}$ for some *k*. Then, by continuity and the fact that $T > 0$, it is necessary that there is some $\tau \in (0,T)$ such that

$$\begin{aligned} \int _{T-\tau }^{T}K_{bk}'(s)ds > \int _{T-\tau }^{T}{\tilde{K}}_{bk}'(s)ds \end{aligned}$$

which means that

$$\begin{aligned} K_{bk}(T-\tau ) < {\tilde{K}}_{bk}(T-\tau ) \end{aligned}$$

which is a contradiction to the definition of *T*. Thus, it is necessary that

43 $$\begin{aligned} K_{bk}'(T) \le {\tilde{K}}_{bk}'(T)\quad \forall k \in \{1,...,n\}. \end{aligned}$$

Dividing (42) by $\beta _{bk}^1/\mu _k^1$ and (43) by $\beta _{bk}^1$ shows that the inequality system (9)—(15) holds for each *k* (as Lemmas B.2–B.4 hold for any group) and so, following Lemma B.6 and the previous work of this proof, it is necessary that

$$\begin{aligned} W_k(t) = {\tilde{W}}_k(t) \quad \forall t \in [0,T] \end{aligned}$$

This holds for each *k* and hence the proof is complete. $\square$

### Lemma B.8

#### Lemma B.8

Define functions $\Delta ^f_i$ to be

$$\begin{aligned} \Delta ^f_i(t):= f_i(T+t) - {\tilde{f}}_i(T+t) \quad \text {for} \quad f \in \{S,I,R,S^V,I^V,R^V,W\} \end{aligned}$$

and suppose that

44 $$\begin{aligned} \Delta ^f_i(0) = 0 \quad \forall f \in \{S,I,R,S^V,I^V,R^V,W\}. \end{aligned}$$

Suppose further that the $U_i(t)$ are right-continuous step functions. Then, for $t \in [0,\delta ]$ in the limit $\delta \rightarrow 0$, and for any $x,y \in \Re$

$$\begin{aligned}{} & {} \frac{x}{\mu _i^1}\Delta ^R_i + \frac{y}{\mu _i^2} \Delta ^{R^V}_i = \frac{t^3S_i(T)(U_i(T)-{\tilde{U}}_i(T))}{6(N_i-W_i(T))}\\ {}{} & {} \left[ x\sum _{j=1}^n(K_{ij}'(T)) -y\sum _{j=1}^n(L'_{ij}(T))\right] + O(\delta ^4). \end{aligned}$$

#### Proof

As the $U_i(t)$ are step functions, for sufficiently small $\delta$, they are constant on the interval $[T,T+\delta ]$, so this will be assumed. Note that, for any $i \in \{1,...,n\}$ and any $t\ge 0$

$$\begin{aligned} \left| \frac{dS_i}{dt}(t)\right|&\le \left| \sum _{j=1}^n S_i(t)\beta ^{1}_{ij}I_j(t)\right| + \left| \frac{S_i(t)}{N_i - W_i(t)}U_i(t)\right| \\&\le \left| \sum _{j=1}^n N_i\beta ^{1}_{ij}N_j\right| + + \left| 1 \times U_i(t)\right| \\&\le U_i(T) + C, \end{aligned}$$

where the constant term, *C*, is independent of *t* and the vaccination policy. Note the second line follows from the fact that, as $W_i(t) < N_i$,

$$\begin{aligned} \frac{S_i(t)}{N_i - W_i(t)} = \frac{S_i(0)}{N_i}\exp \left[ -\sum _{j=1}^nK_{ij}(t)\right] \le 1. \end{aligned}$$

Similarly, one can show (by increasing the constant *C* if necessary) that

$$\begin{aligned} \left| \frac{dS^V_i}{dt}(t)\right|&\le U_i(T) + C \\ \left| \frac{dI^V_i}{dt}(t)\right| , \left| \frac{dR^V_i}{dt}(t)\right| , \left| \frac{dI_i}{dt}(t)\right| ,\left| \frac{dR_i}{dt}(t)\right|&\le C\\ \left| \frac{dW_i}{dt}(t)\right|&\le U_i(T). \end{aligned}$$

Then, for $t \in (0,\delta )$ and $f \in \{S,I,R,S^V,I^V,R^V,W\}$

$$\begin{aligned} \vert f_i(T+t) - f_i(T)\vert = \bigg \vert \int _T^{T+t}\frac{df_i}{dt}(s)ds\bigg \vert \le (C+U_i(T))\delta \end{aligned}$$

so that, in particular

45 $$\begin{aligned} f_i(T+t) = f_i(T) + O(\delta ) \quad \forall f \in \{S,I,R,S^V,I^V,R^V,W\}. \end{aligned}$$

Now,

$$\begin{aligned} \frac{d\Delta ^S_i}{dt} = - \sum _{j=1}^n (K_{ij}S_i - {\tilde{K}}_{ij}{\tilde{S}}_j) + \frac{S_iU_i}{N_i - W_i} -\frac{{\tilde{S}}_i{\tilde{U}}_i}{N_i - {\tilde{W}}_i}. \end{aligned}$$

Using (44) and (45), this equation linearises to

$$\begin{aligned} \frac{d\Delta ^S_i}{dt}(t) = \frac{S_i(T)(U_i(t+T)-{\tilde{U}}_i(t+T))}{N_i-W_i(T)} + O(\delta ). \end{aligned}$$

Noting that

$$\begin{aligned} U_i(t+T)-{\tilde{U}}_i(t+T) = U_i(T)-{\tilde{U}}_i(T) \quad \forall t \in [0,\delta ] \end{aligned}$$

this means that

$$\begin{aligned} \frac{d\Delta ^S_i}{dt} = \frac{S_i(T)(U_i(T)-{\tilde{U}}_i(T))}{N_i-W_i(T)} + O(\delta ) \end{aligned}$$

and so (for $t < \delta$)

$$\begin{aligned} \Delta ^S_i(t) = t\frac{S_i(T)(U_i(T)-{\tilde{U}}_i(T))}{N_i-W_i(T)} + O(\delta ^2). \end{aligned}$$

Now, one can linearise the equation for $\Delta ^I_i$. Note that

$$\begin{aligned} \frac{d\Delta ^I_i}{dt} = \sum _{j=1}^n(K_{ij}'S_i - {\tilde{K}}_{ij}'{\tilde{S}}_i) + \mu _i^1 (I_i - {\tilde{I}}_{i}) \end{aligned}$$

and so, with

$$\begin{aligned} I_i(t+T) = I_i(T) + O(\delta ) \end{aligned}$$

and similar expressions for other variables,

$$\begin{aligned} \frac{d\Delta ^I_i}{dt} = O(\delta ) \Rightarrow \Delta _i^I(t) = O(\delta ^2) \quad \text {for }t < \delta \end{aligned}$$

Now, one can linearise in a different way. Note that

$$\begin{aligned} {\tilde{I}}_i(T+t) = I_i(T+t) + O(\delta ^2)\quad \text {and} \quad {\tilde{I}}^V_i(T+t) = I^V_i(T+t) + O(\delta ^2) \end{aligned}$$

so

$$\begin{aligned} {\tilde{K}}_{ij}'(T+t) = K_{ij}'(T+t) + O(\delta ^2). \end{aligned}$$

Thus,

$$\begin{aligned} \frac{d\Delta ^I_i}{dt}(T+t)&= \sum _{j=1}^n(K'_{ij}(T+t)S_i(T+t) - {\tilde{K}}_{ij}'(T+t){\tilde{S}}_i(T+t)) + \mu _i^1 \Delta _i^I(T+t) + O(\delta ^2)\\&= \Delta ^S_i(t)\sum _{j=1}^n(K'_{ij}(T+t)) + O(\delta ^2)\\&= t\frac{S_i(T)(U_i(T)-{\tilde{U}}_i(T))}{N_i-W_i(T)}\sum _{j=1}^n(K_{ij}'(T) + O(\delta )) + O(\delta ^2)\\&= t\frac{S_i(T)(U_i(T)-{\tilde{U}}_i(T))}{N_i-W_i(T)}\sum _{j=1}^n(K_{ij}'(T)) + O(\delta ^2) \end{aligned}$$

and hence

$$\begin{aligned} \Delta ^I_i = \frac{t^2}{2}\frac{S_i(T)(U_i(T)-{\tilde{U}}_i(T))}{N_i-W_i(T)}\sum _{j=1}^n(K_{ij}'(T)) + O(\delta ^3). \end{aligned}$$

Thus,

$$\begin{aligned} \frac{d\Delta ^R_i}{dt} = \Delta ^I_i\mu _i^1 \Rightarrow \Delta ^R_i(t) = \frac{\mu _i^1t^3}{6}\frac{S_i(T)(U_i(T)-{\tilde{U}}_i(T))}{N_i-W_i(T)}\sum _{j=1}^n(K_{ij}'(T)) + O(\delta ^4). \end{aligned}$$

Now, note that

$$\begin{aligned} \frac{d(\Delta ^S_i + \Delta ^{S^V}_i)}{dt} = O(\delta ) \end{aligned}$$

as this derivative has no explicit dependence on *U*. Thus, in particular,

$$\begin{aligned} \Delta ^S_i + \Delta ^{S^V}_i = O(\delta ^2) \end{aligned}$$

and so

$$\begin{aligned} \Delta ^{S^V}_i = -t\frac{S_i(T)(U_i(T)-{\tilde{U}}_i(T))}{N_i-W_i(T)} + O(\delta ^2). \end{aligned}$$

Then, as before (as the equation for $\frac{dI_i}{dt}$ is the same as that for $\frac{dI^V_i}{dt}$, but with $S^V_i$ instead of $S_i$, $\mu _i^1$ instead of $\mu _i^2$ and $K_{ij}$ instead of $L_{ij}$)

$$\begin{aligned} \frac{d\Delta ^{I^V}_i}{dt}(T+t)= -t\frac{S_i(T)(U_i(T)-{\tilde{U}}_i(T))}{N_i-W_i(T)}\sum _{j=1}^n(L_{ij}'(T)) + O(\delta ^2), \end{aligned}$$

which means

$$\begin{aligned} \Delta ^{I^V}_i = -\frac{t^2}{2}\frac{S_i(T)(U_i(T)-{\tilde{U}}_i(T))}{N_i-W_i(T)}\sum _{j=1}^n(L_{ij}'(T)) + O(\delta ^3) \end{aligned}$$

and hence

$$\begin{aligned} \Delta ^{R^V}_i(t) = -\frac{\mu _i^2t^3}{6}\frac{S_i(T)(U_i(T)-{\tilde{U}}_i(T))}{N_i-W_i(T)}\sum _{j=1}^n(L_{ij}'(T)) + O(\delta ^4). \end{aligned}$$

Thus,

$$\begin{aligned}&\frac{x}{\mu _i^1}\Delta ^R_i + \frac{y}{\mu _i^2} \Delta ^{R^V}_i = \frac{t^3S_i(T)(U_i(T)-{\tilde{U}}_i(T))}{6(N_i-W_i(T))}\\ {}&\left[ x\sum _{j=1}^n(K_{ij}'(T)) -y\sum _{j=1}^n(L'_{ij}(T))\right] + O(\delta ^4) \end{aligned}$$

as required. $$\square $$

### Lemma B.9

#### Lemma B.9

Suppose that

$$\begin{aligned} T:= \inf \left\{ t: K_{ij}(t) \ge {\tilde{K}}_{ij} \quad \text {or} \quad L_{ij}(t) \ge {\tilde{L}}_{ij}(t) \quad \text {for some } i,j \in \{1,...,n\} \right\} \end{aligned}$$

exists. Define functions $\Delta ^f_i$ to be

$$\begin{aligned} \Delta ^f_i(t):= f_i(T+t) - {\tilde{f}}_i(T+t) \quad \text {for} \quad f \in \{S,I,R,S^V,I^V,R^V,W\} \end{aligned}$$

and suppose that

$$\begin{aligned} \Delta ^f_i(0) = 0 \quad \forall f \in \{S,I,R,S^V,I^V,R^V,W\}. \end{aligned}$$

Suppose further that the $U_i(t)$ are right-continuous step functions, $\Pi = \{1,...,n\}$ and that

$$\begin{aligned} \beta _{ij}^1> \beta _{ij}^3> 0 \quad \text {and} \quad I_i(0) > 0 \quad \forall i,j \in \{1,...,n\}. \end{aligned}$$

Then,

$$\begin{aligned} \sum _{j=1}^nK_{ij}(t) \ge \sum _{j=1}^n{\tilde{K}}_{ij}(t) \quad \forall t \in [0,T+\delta ] \end{aligned}$$

and

$$\begin{aligned} \sum _{j=1}^nL_{ij}(t) \ge \sum _{j=1}^n{\tilde{L}}_{ij}(t) \quad \forall t \in [0,T+\delta ], \end{aligned}$$

for sufficiently small $\delta$.

#### Proof

By Lemma B.8, with $x = \beta _{li}^1$ and $y = \beta _{li}^2$ for some $l \in \{1,...,n\}$

$$\begin{aligned}{} & {} \frac{\beta _{li}^1}{\mu _i^1}\Delta ^R_i + \frac{\beta _{li}^2}{\mu _i^2} \Delta ^{R^V}_i = \frac{t^3S_i(T)(U_i(T)-{\tilde{U}}_i(T))}{6(N_i-W_i(T))}\\ {}{} & {} \left[ \beta _{li}^1\sum _{j=1}^n(K_{ij}'(T)) -\beta _{li}^2\sum _{j=1}^n(L'_{ij}(T))\right] + O(\delta ^4). \end{aligned}$$

Now, as $\beta _{li}^1 \ge \beta _{li}^2$, $\beta _{li}^1 > 0$ and $K_{ij}'(t)$ and $L'_{ij}(t)$ are non-negative

46 $$\begin{aligned} \beta _{li}^1\sum _{j=1}^n(K_{ij}'(T)) -\beta _{li}^2\sum _{j=1}^n(L'_{ij}(T)) \le 0 \Rightarrow \sum _{j=1}^n(K_{ij}'(T)) \le \sum _{j=1}^n(L_{ij}'(T)). \end{aligned}$$

Noting that

$$\begin{aligned} K_{ij}'(T) \ge L'_{ij}(T) \quad \forall j \in \{1,...,n\}, \end{aligned}$$

(46) requires

$$\begin{aligned} K_{ij}'(T) = L'_{ij}(T) \quad \forall j \in \{1,...,n\} \end{aligned}$$

which, from the definitions of $K'$ and $L'$ requires

$$\begin{aligned} \beta ^1_{ij}I_j(T) +\beta ^2_{ij}I^V_j(T) = \beta ^3_{ij}I_j(T) +\beta ^4_{ij}I^V_j(T). \end{aligned}$$

Thus, as $I_j(T) > 0$ (as $\Pi \in \{1,...,n\}$) and $\beta ^2_{ij}I^V_j(T) \ge \beta ^4_{ij}I^V_j(T)$, it is necessary that

$$\begin{aligned} \beta ^1_{ij} \le \beta ^3_{ij}, \end{aligned}$$

which is a contradiction. Thus,

$$\begin{aligned} \beta ^1_{ij}\sum _{j=1}^n(K_{ij}'(T)) -\beta _{ij}^2\sum _{j=1}^n(L'_{ij}(T)) > 0 \end{aligned}$$

which means

$$\begin{aligned} S_i(T)U_i(T) < S_i(T){\tilde{U}}_i(T) \Rightarrow \frac{\beta ^1_{ij}}{\mu _i^1}\Delta ^R_i + \frac{\beta _{ij}^2}{\mu _i^2} \Delta ^{R^V}_i= -C \delta ^3 + O(\delta ^4) \end{aligned}$$

for some positive constant *C*. Now, if

$$\begin{aligned} S_i(T)U_i(T) > S_i(T){\tilde{U}}_i(T) \end{aligned}$$

then, necessarily, $U_i(T) > {\tilde{U}}_i(T)$. Thus, as $\Delta ^W_i(0) = 0$, one will have

$$\begin{aligned} W_i(T+t) > {\tilde{W}}_i(T+t) \end{aligned}$$

for sufficiently small *t*, which is a contradiction. Moreover, if

$$\begin{aligned} S_i(T)U_i(T) = S_i(T){\tilde{U}}_i(T) \quad \forall i \in \{1,...n\} \end{aligned}$$

then the vaccination policies are the same in the interval $[T,T+\delta ]$, as for each *i*, either $S_i(T) = 0$ (in which case there is no more vaccination in group *i* so $U_i(T) = {\tilde{U}}_i(T) = 0$) or $U_i(T) = {\tilde{U}}_i(T)$. Thus, the disease trajectories are the same, which contradicts the definition of *T*, as then $K_{ij}(T+t) = {\tilde{K}}_{ij}(T+t)$ and $L_{ij}(T+t) = {\tilde{L}}_{ij}(T+t)$ for all $t \in [0,\delta ]$.

Now, note that

$$\begin{aligned} \sum _{i=1}^nK_{li}(t) - \sum _{i=1}^n{\tilde{K}}_{li}(t) = \sum _{i=1}^n\bigg (\frac{\beta ^1_{li}}{\mu ^1_i}\Delta ^R_i + \frac{\beta {li}^2}{\mu _i^2}\Delta ^{R^V}_i\bigg ) = - \sum _{i=1}^nE_i\delta ^3 + O(\delta ^4), \end{aligned}$$

where $E_i > 0$ if $U_i(T) < {\tilde{U}}_i(T)$ and $E_i = 0$ otherwise. Thus, in particular

$$\begin{aligned} \sum _{i=1}^n E_i > 0 \end{aligned}$$

and hence

$$\begin{aligned} \sum _{i=1}^nK_{li}(t) - \sum _{i=1}^n{\tilde{K}}_{li}(t)= - \sum _{i=1}^nE_i\delta ^3 + O(\delta ^4) < 0 \end{aligned}$$

for sufficiently small $\delta$. Thus,

$$\begin{aligned} \sum _{j=1}^nK_{ij}(t) \ge \sum _{j=1}^n{\tilde{K}}_{ij}(t) \quad \forall t \in [0,T+\delta ] \end{aligned}$$

and, by identical arguments (using $x = \beta _{li}^3$ and $y = \beta _{li}^4$ in Lemma B.8)

$$\begin{aligned} \sum _{j=1}^nL_{ij}(t) \ge \sum _{j=1}^n{\tilde{L}}_{ij}(t) \quad \forall t \in [0,T+\delta ] \end{aligned}$$

as required. $$\square $$

### Lemma B.10

#### Lemma B.10

Suppose that

$$\begin{aligned} T:= \inf \left\{ t: K_{ij}(t)< {\tilde{K}}_{ij}(t) \quad \text {or} \quad L_{ij}(t) < {\tilde{L}}_{ij}(t) \quad \text {for some } i,j \in \{1,...,n\} \right\} . \end{aligned}$$

Then, for any $\delta > 0$, there exists some $t \in (T,T+\delta )$ and some real parameters $0\le \kappa \le \eta \le 1$ such that

$$\begin{aligned} R_b(t) + \kappa R^V_b(t) < {\tilde{R}}_b(t) + \kappa {\tilde{R}}^V_b(t) \quad \text {and} \quad I_b(t) + \eta I^V_b(t) \le {\tilde{I}}_b(t) + \eta {\tilde{I}}^V_b(t). \end{aligned}$$

#### Proof

Firstly, note that by the definition of *T*, for each $\delta > 0$, there must exist $i,j \in \{1,...,n\}$ and $t \in (0,\delta )$ such that

$$\begin{aligned} K_{ij}(T+t)< {\tilde{K}}_{ij}(T+t) \quad \text {or} \quad L_{ij}(T+t) < {\tilde{L}}_{ij}(T+t). \end{aligned}$$

That is, there is some $b \in \{1,..,n\}$ such that

47 $$\begin{aligned} R_b(T+t) + \kappa R^V_b(T+t) < {\tilde{R}}_b(T+t) + \kappa {\tilde{R}}^V_b(T+t) \end{aligned}$$

where

$$\begin{aligned} \kappa \le \frac{\mu _b^1}{\mu _b^2}. \end{aligned}$$

Note that

$$\begin{aligned} \mu _b^1 I_b(t) + \kappa \mu _b^2 I_b^V(t) = \frac{d}{dt}\left( R_b(t) + \kappa R^V_b(t)\right) . \end{aligned}$$

Now, define

$$\begin{aligned} \Delta ^f_i(t):= f_i(T+t) - {\tilde{f}}_i(T+t) \quad \forall f \in \{I,I^V,R,R^V\} \end{aligned}$$

and

$$\begin{aligned} \tau := \sup \{s \in [0,t]: \Delta ^R_b(s) + \kappa \Delta ^{R^V}_b(s) \ge 0\} \end{aligned}$$

which exists as $\Delta ^R_b(0) + \kappa \Delta ^{R^V}_b(0)=0$. Note that $\tau < t$ by (47). Note also that by continuity, it is necessary that

$$\begin{aligned} \Delta ^R_b(\tau ) + \kappa \Delta ^{R^V}_b(\tau ) = 0. \end{aligned}$$

Now, by the mean value theorem (as $\Delta ^R_b + \kappa \Delta ^{R^V}_b$ is continuously differentiable), there exists an *s* in the non empty interval $(\tau ,t)$ such that

$$\begin{aligned} \mu _1^b \Delta ^I_b(s) + \kappa \mu _b^2 \Delta ^{I^V}_b(s)&= \frac{1}{t-\tau }\bigg [(\Delta ^R_b(t) + \kappa \Delta ^{R^V}_b(t)) - (\Delta ^R_b(\tau ) + \kappa \Delta ^{R^V}_b(\tau ))\bigg ] \\&= \frac{1}{t-\tau }\bigg [\Delta ^R_b(t) + \kappa \Delta ^{R^V}_b(t)\bigg ]\\&< 0 \end{aligned}$$

while also

$$\begin{aligned} \Delta ^R_b(s) + \kappa \Delta ^{R^V}_b(s) < 0, \end{aligned}$$

by definition of $\tau$. Thus, defining $\eta := \kappa \frac{\mu _b^2}{\mu _b^1} \le 1$,

$$\begin{aligned} \Delta ^R_b(s) + \kappa \Delta ^{R^V}_b(s) < 0 \quad \Delta ^I_b(s) +\eta \Delta ^{I^V}_b(s) \ge 0 \quad \text {and} \quad 0\le \kappa \le \eta \le 1 \end{aligned}$$

as required. $$\square $$

### Lemma B.11

#### Lemma B.11

Consider two non-negative functions *A*(*t*) and *B*(*t*) such that *B*(*t*) is non-decreasing and differentiable with a Lebesgue integrable derivative $B'(t)$ satisfying

$$\begin{aligned} \int _0^tB'(s)ds = B(t)-B(0) \quad \forall t \ge 0. \end{aligned}$$

Suppose further that for each $T\ge 0$, one can partition the interval [0, *T*] into a finite number of subintervals $S^A_1,...,S^A_m$ and $S^B_1,...,S^B_k$ such that

$$\begin{aligned} s \in \bigcup _{i = 1}^mS^A_i \Leftrightarrow A(s) > B'(s) \end{aligned}$$

Then, there exists a unique function $\chi (t)$ for $t \ge 0$ such that

$$\begin{aligned} \chi (t):= \left\{ \begin{matrix} A(t) &{} \text {if} \quad \int _0^t\chi (s)ds < B(t) \\ \min (A(t),B'(t)) &{} \text {if} \quad \int _0^t\chi (s)ds \ge B(t) \end{matrix}\right. \end{aligned}$$

#### Proof

$\chi$ can be constructed for each of the subintervals $S^A_i$ and $S_i^B$. Note first that,

$$\begin{aligned} t \in S^B_i \Rightarrow B'(t) \ge A(t) \Rightarrow \chi (t) = A(t) \end{aligned}$$

Now, suppose that $t \in S^A_i$ for some *i*. Then, as $S^A_i$ is an interval, one can suppose $S^A_i = [c_i,d_i]$. Define

$$\begin{aligned} \tau := \inf \bigg (\bigg \{s \in S^A_i: B(s) \le \int _0^{c_i}\chi (u)du + \int _{c_i}^sA(u)du\bigg \} \cup \{d_i\}\bigg ). \end{aligned}$$

If $\tau = d_i$, then one has (uniquely) $\chi (t) = A(t)$ in $S^A_i$. Otherwise, one has (again uniquely)

$$\begin{aligned} \chi (t) = A(t) \quad \forall t \in [c_i,\tau ] \quad \text {and} \quad \chi (t) = B'(t) \quad \forall t \in [\tau ,d_i] \end{aligned}$$

Uniqueness can be demonstrated as follows. If $\chi (t) =B'(t)$ for some $t \in [c_i,\tau ]$, then it is necessary (as $A(t) > B'(t)$ so $\chi (t) \ne A(t)$ in this case)

$$\begin{aligned} \int _{0}^t\chi (s) \ge B(t) \end{aligned}$$

As $A(t) \ge B'(t)$ in $S^A_i$, so $\chi (t)$ is bounded by *A*(*t*), the previous inequality can be extended to give

$$\begin{aligned} B(t) \le \int _{0}^t\chi (s) \le \int _0^{c_i}\chi (u)du + \int _{c_i}^sA(u)du \end{aligned}$$

which contradicts the definition of $\tau$. A similar argument stands to prove uniqueness in $[\tau ,d_i]$.

Thus, $\chi$ is uniquely defined in each of the finite number of intervals and hence in [0, *T*] for each *T* and hence, it is uniquely defined for all *t* as required. $\square$

## Results on the SIR Equations

This section presents a variety of results on the SIR equations which are used in the proofs of the theorems in this paper. Many of them are well-known and widely used in the literature, but this appendix aims to provide a source of formal definitions and proofs of these results.

Before the results can be proved, it is necessary to establish two lemmas on differential equations.

### Lemma C.1

#### Lemma C.1

Suppose that *H*(*t*) is a continuous non-negative $n \times n$ matrix for $t \ge 0$ and that ${\varvec{a}} \in \Re ^n$. Then, suppose that a function ${\varvec{u}}: \Re \rightarrow \Re ^n$ satisfies

$$\begin{aligned} {\varvec{u}}(t) \le {\varvec{a}} + \int _0^t H(s) {\varvec{u}}(s)ds \quad \forall t \ge 0. \end{aligned}$$

Then,

$$\begin{aligned} {\varvec{u}}(t) \le \left( 1 + \int _0^t V(t,s)H(s)ds\right) {\varvec{a}}, \end{aligned}$$

where the matrix *V*(*t*, *s*) satisfies

$$\begin{aligned} V(t,s) = I_n + \int _s^tH(k)V(k,s)dk \end{aligned}$$

and $I_n$ is the $n \times n$ identity matrix.

#### Proof

This theorem is a special case of the theorem proved in Chandra and Davis (1976) where (in the notation of Chandra and Davis 1976), *x*, *y* and *z* have been replaced by *t*, *s* and *k* respectively, *G*(*t*) has been set to be the identity matrix and $x^0$ has been set to zero. $\square$

### Lemma C.2

#### Lemma C.2

Consider a continuous, time-dependent, matrix *A*(*t*) which satisfies

$$\begin{aligned} A(t)_{ij} \ge 0 \quad \forall t \ge 0 \quad \text {and} \quad \forall i \ne j \end{aligned}$$

and a constant matrix *B* that satisfies

$$\begin{aligned} B_{ij} \ge 0 \quad \forall t \ge 0 \quad \text {and} \quad \forall i \ne j. \end{aligned}$$

Then, suppose that each element of *A*(*t*) is non-increasing with *t* and that

$$\begin{aligned} A(t)_{ij} \ge B_{ij} \quad \forall t \ge 0 \quad \text {and} \quad \forall i \ne j. \end{aligned}$$

Moreover, define a non-negative initial condition ${\varvec{v}}$ and suppose that ${\varvec{y}}$ and ${\varvec{z}}$ solve the systems

$$\begin{aligned} \frac{d{\varvec{y}}}{dt} = A(t) {\varvec{y}} \quad \text {and} \quad \frac{d{\varvec{z}}}{dt} = B {\varvec{z}} \end{aligned}$$

with

$$\begin{aligned} {\varvec{y}}(0) = {\varvec{z}}(0) = {\varvec{v}} \ge {\varvec{0}}. \end{aligned}$$

Then,

$$\begin{aligned} {\varvec{y}}(t) \ge {\varvec{z}}(t) \ge {\varvec{0}} \quad \forall t \ge 0. \end{aligned}$$

#### Proof

To begin, define

$$\begin{aligned} \mu := \min _i \bigg (B_{ii}\bigg ) \end{aligned}$$

so that, defining

$$\begin{aligned} A^*(t):= A(t) + \mu I \quad \text {and} \quad B^*:= B + \mu I, \end{aligned}$$

where *I* is the identity matrix, $A^*$ and $B^*$ are non-negative matrices. Moreover, note that

$$\begin{aligned} \frac{d{\varvec{y}}}{dt} + \mu {\varvec{y}} = A^*(t) {\varvec{y}} \end{aligned}$$

and so

$$\begin{aligned} e^{-\mu t} \frac{d}{dt} \left( e^{\mu t} {\varvec{y}}\right) = A^*(t) {\varvec{y}}. \end{aligned}$$

Thus, define

$$\begin{aligned} {\varvec{y}}^*(t):= e^{\mu t} {\varvec{y}}(t) \end{aligned}$$

so

$$\begin{aligned} \frac{d{\varvec{y}}^*}{dt} = A^*(t) {\varvec{y}}^*. \end{aligned}$$

Similarly, defining

$$\begin{aligned} {\varvec{z}}^*(t):= e^{\mu t} {\varvec{z}}(t) \end{aligned}$$

gives

$$\begin{aligned} \frac{d{\varvec{z}}^*}{dt} = B{\varvec{z}}^* \end{aligned}$$

while, moreover,

$$\begin{aligned} {\varvec{y}}^* \ge {\varvec{z}}^* \Leftrightarrow {\varvec{y}} \ge {\varvec{z}} \quad \text {and} \quad {\varvec{z}}^* \ge {\varvec{0}} \Leftrightarrow {\varvec{z}} \ge {\varvec{0}}. \end{aligned}$$

Thus, it is simply necessary to prove that the results of this lemma hold when *A*(*t*) and *B* are non-negative matrices.

Now, it is helpful to note that, as the off-diagonal entries of *A*(*t*) and *B* are non-negative, the two differential systems are totally positive (Schwarz 1970). Thus, in particular, as ${\varvec{v}}$ is non-negative,

$$\begin{aligned} {\varvec{y}}(t), {\varvec{z}}(t) \ge {\varvec{0}} \quad \forall t \ge 0, \end{aligned}$$

which proves one of the required inequalities. Now, one can also note that

$$\begin{aligned} \frac{d}{dt}\bigg ({\varvec{y}} - {\varvec{z}}\bigg ) = A(t){\varvec{y}} - B{\varvec{z}}. \end{aligned}$$

As *A*(*t*) is assumed to be non-negative, and ${\varvec{y}}$ is non-negative,

$$\begin{aligned} \frac{d}{dt}\bigg ({\varvec{y}} - {\varvec{z}}\bigg ) \ge B({\varvec{y}} - {\varvec{z}}). \end{aligned}$$

Defining $\varvec{\zeta }:= {\varvec{z}} - {\varvec{y}}$ and integrating gives

$$\begin{aligned} \varvec{\zeta }(t) \le \int _0^{t}B(s)\varvec{\zeta }(s)ds, \end{aligned}$$

noting that $\varvec{\zeta } = {\varvec{0}}$. Hence, by Lemma C.1, one has

$$\begin{aligned} \varvec{\zeta }(t) \le {\varvec{0}} \Rightarrow {\varvec{y}} \ge {\varvec{z}} \end{aligned}$$

as required. $$\square $$

### Lemma C.3

#### Lemma C.3

Define the set of functions

$$\begin{aligned} {\mathcal {F}}_i(t):= \bigg \{ S_i(t), I_i(t),R_i(t),S^V_i(t),I^V_i(t),R^V_i(t)\bigg \}. \end{aligned}$$

Then, for all $t \ge 0$ and $i \in \{1,...,n\}$,

$$\begin{aligned} 0 \le f \le N_i \quad \forall f \in {\mathcal {F}}_i(t). \end{aligned}$$

#### Proof

Noting that

$$\begin{aligned} \sum _{f \in {\mathcal {F}}_i(t)} f = N_i, \end{aligned}$$

it is simply necessary to show that (for each *t* and *i*)

$$\begin{aligned} f(t) \ge 0 \quad \forall f(t) \in {\mathcal {F}}_i(t). \end{aligned}$$

Now, note that

$$\begin{aligned} \frac{dS_i}{dt} = -\sum _{j=1}^n(\beta ^1_{ij}I_j+ \beta ^2_{ij} I^V_j)S_i - \frac{U_i(t) S_i}{N_i-W_i(t)}, \end{aligned}$$

which means

$$\begin{aligned} \frac{d}{dt}\bigg (S_i \exp \left[ -\sum _{j=1}^n(\frac{\beta ^1_{ij}}{\mu ^1_j}R_j + \frac{\beta _{ij}^2}{\mu ^2_j} R^V_j) - \ln (N_i - W_i)\right] \bigg ) = 0 \end{aligned}$$

and hence (using the initial conditions)

$$\begin{aligned} S_i(t) = \frac{S_i(0)(N_i-W_i(t))}{N_i}\exp \bigg (-\sum _{j=1}^n\bigg [\frac{\beta ^1_{ij}}{\mu ^1_j}R_j + \frac{\beta _{ij}^2}{\mu ^2_j} R^V_j\bigg ]\bigg ). \end{aligned}$$

As $W_i(t) \le N_i$ by construction, this means that

$$\begin{aligned} S_i(t) \ge 0 \quad \text {as required}. \end{aligned}$$

Now, note that

$$\begin{aligned} \frac{dS^V_i}{dt} = -\sum _{j=1}^n(\beta ^3_{ij}I_j + \beta _{ij}^4 I^V_j)S^V_i + \frac{U_i(t) S_i}{N_i-W_i(t)} \ge -\sum _{j=1}^n(\beta ^3_{ij}I_j + \beta _{ij}^4 I^V_j)S^V_i \end{aligned}$$

so that

$$\begin{aligned} \frac{d}{dt} \bigg ( S^V_i \exp \left[ \sum _{j=1}^n\bigg (\frac{\beta ^3_{ij}}{\mu ^1_j}R_j + \frac{\beta _{ij}^4}{\mu ^2_j} R^V_j\bigg )\right] \bigg )\ge 0, \end{aligned}$$

which means (as $S^V_i(0) = 0$)

$$\begin{aligned} S^V_i(t)\exp \left[ \sum _{j=1}^n\bigg (\frac{\beta ^3_{ij}}{\mu ^1_j}R_j(t) + \frac{\beta _{ij}^4}{\mu ^2_j} R^V_j(t)\bigg )\right] \ge 0 \end{aligned}$$

and hence

$$\begin{aligned} S^V_i(t) \ge 0 \quad \text {as required}. \end{aligned}$$

Now, define the vector

$$\begin{aligned} {\varvec{y}}:= \begin{pmatrix} {\varvec{I}}\\ {\varvec{I}}^V\\ \end{pmatrix} \end{aligned}$$

Then, one can rewrite the equations for $I_i$ and $I^V_i$ in the form

$$\begin{aligned} \frac{d {\varvec{y}}}{dt} = M({\varvec{S}}(t),{\varvec{S}}^V(t)) {\varvec{y}} \end{aligned}$$

for some matrix *M*, where, from the previous results

$$\begin{aligned} M_{ij} \ge 0 \quad \forall i \ne j. \end{aligned}$$

Thus, from Lemma C.2,

$$\begin{aligned} {\varvec{y}}(t) \ge {\varvec{0}} \quad \forall t \ge 0. \end{aligned}$$

Then,

$$\begin{aligned} \frac{dR_i}{dt} = \mu _i^1 I_i \ge 0 \quad \text {so} \quad R_i(t) \ge 0 \end{aligned}$$

and similarly,

$$\begin{aligned} R^V_i(t) \ge 0 \end{aligned}$$

and so the proof is complete. $\square$

### Lemma C.4

#### Lemma C.4

For each *i*,

$$\begin{aligned} \lim _{t \rightarrow \infty }(I_i(t)) = \lim _{t \rightarrow \infty }(I^V_i(t)) = 0. \end{aligned}$$

#### Proof

Firstly, suppose

$$\begin{aligned} \lim _{t \rightarrow \infty } \left( \text {inf}\left\{ I_i(s): s \ge t \right\} \right) = Q, \end{aligned}$$

noting this infimum exists as $I_i$ is bounded below by 0, and the limit exists as the sequence of infima given $s \le t$ is non-decreasing and bounded above by $N_i$. If $Q \ne 0$, there exists some $m > 0$ and some *t* such that for all $s \ge t$

$$\begin{aligned} I_i(s) \ge m \Rightarrow \frac{dR_i}{dt}(s) \ge m\mu ^1_i \Rightarrow R_i\left( t + \frac{2N_i}{m\mu ^1_i}\right) > N_i \end{aligned}$$

which contradicts Lemma C.3. Thus, $Q = 0$ and so there exists some sequence $t_n$ such that

48 $$\begin{aligned} \lim _{n \rightarrow \infty }(t_n) = \infty \quad \text {and} \quad \lim _{n \rightarrow \infty }(I(t_n)) = 0. \end{aligned}$$

Now note that $S_i(t)$ is non-increasing and bounded and that $R_i(t)$ and $(S^V_i(t) + I^V_i(t) + R^V_i(t))$ are non-decreasing and bounded. Thus, their limits as $t \rightarrow \infty$ must exist and be finite, so in particular

$$\begin{aligned} \lim _{t \rightarrow \infty }(I_i(t)) = \lim _{t \rightarrow \infty }(N_i - S_i(t) - R_i(t) - S^V_i(t) - I^V_i(t) - R^V_i(t)) \end{aligned}$$

must exist. Thus, by (48), the only possible limit is 0 so

$$\begin{aligned} \lim _{t \rightarrow \infty }(I_i(t)) = Q = 0 \end{aligned}$$

as required. By noting that $S_i(t) + S^V_i(t)$ is non-increasing and that $I_i(t) + R_i(t)$ and $R^V_i(t)$ are non-decreasing, an identical argument shows that

$$\begin{aligned} \lim _{t \rightarrow \infty }(I^V_i(t)) = 0. \end{aligned}$$

$$\square $$

### Lemma C.5

#### Lemma C.5

Suppose that $I_i(t) > 0$ for some $t\ge 0$ and some $i \in \{1,...,n\}$. Then,

$$\begin{aligned} I_i(s)> 0 \quad \forall s > t. \end{aligned}$$

An analogous result holds for $I^V_i(t)$.

#### Proof

Note that

$$\begin{aligned} \frac{dI_i}{dt} \ge -\mu _i^1I_i \end{aligned}$$

and so

$$\begin{aligned} \frac{d}{dt}\left( e^{\mu _i^1 t}I_i(t)\right) \ge 0 \end{aligned}$$

which means, for any $s > t$

$$\begin{aligned} e^{\mu _i^1 s}I_i(s) \ge e^{\mu _i^1 t}I_i(t) \end{aligned}$$

and hence

$$\begin{aligned} I_i(s) > 0 \end{aligned}$$

as required. The same argument then works for $I^V_i(t)$ as well (with a $\mu _i^2$ instead of a $\mu _i^1$). $\square$

### Lemma C.6

#### Lemma C.6

Define

$$\begin{aligned} \Pi := \left\{ i: \exists t\ge 0 \quad \text {s.t.} \quad I_i(t)> 0 \quad \text {or} \quad I_i^V(t) > 0\right\} . \end{aligned}$$

Moreover, define

$$\begin{aligned} \Pi ^0:= \left\{ i: I_i(0) > 0 \right\} \end{aligned}$$

and the *n* by *n* matrix *M* by

$$\begin{aligned} M_{ij} = S_i(0)\beta ^1_{ij}. \end{aligned}$$

Then, define the connected component *C* of $\Pi ^0$ in *M* as follows. The index $i \in \{1,...,n\}$ belongs to *C* if any only if there is some sequence $a_1,..., a_k$ such that

$$\begin{aligned} a_j\in & {} \{1,...,n\} \quad \forall j \in \{1,...,k\}, \\{} & {} M_{a_1,a_2}M_{a_2,a_3}...,M_{a_{k-1}a_k} > 0 \end{aligned}$$

and

$$\begin{aligned} a_1 = i\quad \text {and} \quad a_k \in \Pi ^0. \end{aligned}$$

Then,

**(a)** $$ i \in C \Rightarrow I_i(t)> 0 \quad \forall t > 0$$.

**(b)** $$\Pi = C \cup \Pi ^0$$.

Thus, in particular,

$$\begin{aligned} i \in C\cup \Pi ^0 = \Pi \Leftrightarrow I(t)> 0 \quad \forall t > 0. \end{aligned}$$

#### Proof

**(a):** The proof will proceed by induction. For $k \ge 1$, define $P^k$ is the set of elements of *C* that are connected to an element of $\Pi ^0$ by a sequence of length at most *k*. Then, note that

$$\begin{aligned} P^{k} \subseteq P^{k+1} \quad \forall k \ge 1 \end{aligned}$$

and

$$\begin{aligned} P^{n^2} = C \end{aligned}$$

as there are $n^2$ elements in *M*. (Thus, if $i \in C$ then there must be a sequence of length at most $n^2$ connecting *i* with an element in $\Pi ^0$ as any loops can be ignored.)

The inductive hypothesis is that

$$\begin{aligned} i \in P^k \Rightarrow I_i(t)> 0 \quad \forall t > 0. \end{aligned}$$

The explanation of the base case will be left until the end of the proof. Suppose that this claim holds for some $k \ge 0$. If $P^{k+1} = P^k$, then

$$\begin{aligned} i \in P^{k+1} \Rightarrow i \in P^{k} \Rightarrow I_i(t)> 0 \quad \forall t > 0 \end{aligned}$$

and so the inductive step is complete. Otherwise, consider any $i \in P^{k+1}/P^k$. Then, there exists some *j* such that

$$\begin{aligned} M_{ij} > 0 \quad \text {and} \quad j \in P^k. \end{aligned}$$

Thus, by continuity, for sufficiently small $\tau$,

$$\begin{aligned} t < \tau \Rightarrow S_i(t)\beta ^1_{ij} > 0 \end{aligned}$$

and indeed, by Boundedness Theorem, there exists some $\chi > 0$ such that

$$\begin{aligned} S_i(t)\beta ^1_{ij} > \chi \quad \forall t \in [0,\tau ]. \end{aligned}$$

Now, choose any $\epsilon \in [0,\tau ]$. By Boundedness Theorem, $I_i(t)$ achieves is bounded and achieves its maximum, $\theta _{\epsilon }$ in the interval $[0,\epsilon ]$. Moreover, $\theta _{\epsilon } > 0$ as $I_i(t) > 0$ in $(0,\epsilon )$ by assumption. Thus, by continuity, there exists some non-empty region $(\delta _{\epsilon },\Delta _{\epsilon })$ such that

$$\begin{aligned} t \in (\delta _{\epsilon },\Delta _{\epsilon }) \Rightarrow I_i(t) > \frac{\theta _{\epsilon }}{2}. \end{aligned}$$

Thus, in particular

$$\begin{aligned} \int _0^{\epsilon }S_i(t)\beta ^1_{ij}I_j(t) dt \ge \chi \int _{\delta _{\epsilon }}^{\Delta _{\epsilon }}I_j(t) dt \ge \frac{\chi \theta _{\epsilon }}{2}(\Delta _{\epsilon } - \delta _{\epsilon }) > 0. \end{aligned}$$

Now, note that

$$\begin{aligned} \frac{dI_i}{dt} \ge S_i(t)\beta ^1_{ij}I_j(t) - \mu _i^1I_i(t). \end{aligned}$$

Suppose for a contradiction that $I_i(t) = 0$ for all $t \in [0,\epsilon ]$. Then,

$$\begin{aligned} \frac{dI_i}{dt} \ge S_i(t)\beta ^1_{ij}I_j(t) \Rightarrow I_i(\epsilon ) \ge I_i(0) + \frac{\chi M_{\epsilon }}{2}(\Delta _{\epsilon } - \delta _{\epsilon }) \end{aligned}$$

and hence,

$$\begin{aligned} I_i(\epsilon ) > 0, \end{aligned}$$

which is a contradiction. Thus, there exists a $t \in [0,\epsilon ]$ such that $I_i(t) > 0$ and hence, by Lemma C.5,

$$\begin{aligned} I_i(t) > 0 \quad \forall t \in [\epsilon , \infty ). \end{aligned}$$

Thus, as $\epsilon$ was any constant in the region $(0,\tau )$, and $\tau > 0$, this means that

$$\begin{aligned} I_i(t)> 0 \quad \forall t > 0 \end{aligned}$$

as required.

Finally, note that the base case $k=1$ can be proved in exactly the same way, except now $j \in \Pi ^0$ (but this still means that $I_j(t) > 0$ for all $t>0$ by Lemma C.5), and so **(a)** has been proved.

**(b)**: The previous work has shown that

$$\begin{aligned} C \subseteq \Pi . \end{aligned}$$

Hence, as clearly $\Pi ^0 \subseteq \Pi$, this means that

$$\begin{aligned} C \cup \Pi ^0 \subseteq \Pi \end{aligned}$$

and so it suffices to prove that

$$\begin{aligned} \Pi \subseteq C \cup \Pi ^0. \end{aligned}$$

That is, it suffices to prove

$$\begin{aligned} i \notin C \cup \Pi ^0 \Rightarrow I_i(t) =I^V_i(t) = 0 \quad \forall t \ge 0. \end{aligned}$$

To check that this solution satisfies the equations, one notes that, in this case, if $i \notin C \cup \Pi ^0$, then

$$\begin{aligned} \frac{dI_i}{dt}&= \sum _{j=1}^n S_i(t)\beta ^1_{ij}I_j(t) + \sum _{j=1}^n S_i(t)\beta ^2_{ij}I^V_j(t)- \mu I_i(t)\\&= \sum _{j\in C\cup \Pi ^0} S_i(t)\beta ^1_{ij}I_j(t) + \sum _{j\in C\cup \Pi ^0} S_i(t)\beta ^2_{ij}I^V_j(t) \end{aligned}$$

and, similarly,

$$\begin{aligned} \frac{dI^V_i}{dt} = \sum _{j\in C\cup \Pi ^0} S^V_i(t)\beta ^3_{ij}I_j(t) + \sum _{j\in C\cup \Pi ^0} S_i(t)\beta ^4_{ij}I^V_j(t), \end{aligned}$$

as $I_j(t) = I^V_j(t) = 0$ for all $j \notin C\cup \Pi ^0$.

Now, suppose that $i \notin C\cup \Pi ^0$ and $j \in C \cup \Pi ^0$. Then, by definition of *C*, this means that

$$\begin{aligned} M_{ij} = S_i(0)\beta ^1_{ij} = 0 \quad \forall j \in C \cup \Pi ^0 \end{aligned}$$

and hence, as $S_i$ is non-increasing and non-negative

$$\begin{aligned} S_i(t)\beta ^1_{ij} = 0 \quad \forall j \in C \cup \Pi ^0. \end{aligned}$$

Now, as $\beta ^1_{ij}\ge \beta _{ij}^2 \ge 0$, this means that

$$\begin{aligned} S_i(t)\beta ^2_{ij} = 0 \quad \forall j \in C \cup \Pi ^0 \end{aligned}$$

so that

$$\begin{aligned} \sum _{j\in C\cup \Pi ^0} S_i(t)\beta ^1_{ij}I_j(t) + \sum _{j\in C\cup \Pi ^0} S_i(t)\beta ^2_{ij}I^V_j(t)= 0, \end{aligned}$$

which means

$$\begin{aligned} \frac{dI_i}{dt} = 0 \quad \text {as required}. \end{aligned}$$

Moreover, as $S^V_i(0) = 0$, it is necessary that

$$\begin{aligned} (S_i(0) + S^V_i(0))\beta ^1_{ij} = 0\quad \forall j \in C \cup \Pi ^0 \end{aligned}$$

so, as $(S_i + S^V_i)\beta ^1_{ij}$ is non-increasing and non-negative

$$\begin{aligned} (S_i(t) + S^V_i(t))\beta ^1_{ij} = 0\quad \forall j \in C \cup \Pi ^0 \end{aligned}$$

and hence, as $S_i(t)$ is non-negative

$$\begin{aligned} (S^V_i(t))\beta ^1_{ij} = 0\quad \forall j \in C \cup \Pi ^0. \end{aligned}$$

Thus, as $\beta ^1_{ij} \ge \beta ^3_{ij} \ge \beta ^4_{ij} \ge 0$, one has

$$\begin{aligned} \sum _{j\in C\cup \Pi ^0} S^V_i(t)\beta ^3_{ij}I_j(t) + \sum _{j\in C\cup \Pi ^0} S_i(t)\beta ^4_{ij}I^V_j(t) = 0 \end{aligned}$$

and hence

$$\begin{aligned} \frac{dI^V_i}{dt} = 0 \quad \text {as required}. \end{aligned}$$

Then, one can separately solve the system for all $j \in C \cup \Pi ^0$ as the equations will now be independent of any indices $i \notin C \cup \Pi ^0$ (as they only depend on these indices via the $I_i$ and $I_i^V$ terms, which are identically zero). Thus, by the uniqueness of solution, one must have

$$\begin{aligned} i \in C \cup \Pi ^0 \Rightarrow I_i(t) = I_i^V(t) = 0 \quad \forall t \ge 0 \end{aligned}$$

and hence part **(b)** is proved. Thus, the lemma has been proved. $\square$

### Lemma C.7

#### Lemma C.7

Consider a set $C = [a_1,b_1]\times [a_2,b_2]\times ...\times [a_n,b_n]$ that is a Cartesian product of real intervals. Suppose that $f: \Re ^n \rightarrow \Re$ is differentiable with bounded derivatives in *C*. Then, *f* is Lipschitz continuous on *C* - that is, there exists some $L > 0$ such that

$$\begin{aligned} \vert f({\varvec{x}}) - f({\varvec{y}})\vert \le L\sum _{i=1}^n\vert x_i - y_i\vert \quad \forall {\varvec{x}},{\varvec{y}} \in C. \end{aligned}$$

#### Proof

Note that, by assumption, for each *i*,

$$\begin{aligned} \frac{\partial f}{\partial x_i} \quad \text {is bounded in { C}}, \end{aligned}$$

so define the global bound for all *i* to be M. Choose some ${\varvec{x}}, {\varvec{y}} \in C$. Define the points ${\varvec{p}}^k \in C$ for $k = 0,1,...,n$ by

$$\begin{aligned} p^k_i = \left\{ \begin{matrix} y_i &{} \text {if }i \le k\\ x_i &{} \text {otherwise}\\ \end{matrix}\right. \end{aligned}$$

and define the curve $\gamma _i$ to be the straight line joining the point ${\varvec{p}}^{i-1}$ to the point ${\varvec{p}}^{i}$. As *C* is a product of intervals, the $\gamma _i$ lie entirely in *C*.

Define $\Gamma$ to be the union of the curves $\gamma _i$, so that $\Gamma$ joins ${\varvec{p}}^0 = {\varvec{x}}$ to ${\varvec{p}}^n = {\varvec{y}}$. Then

$$\begin{aligned} \vert f({\varvec{x}}) - f({\varvec{y}})\vert&= \bigg \vert \int _{\Gamma }\nabla f \cdot d{\varvec{x}}\bigg \vert \\&= \bigg \vert \sum _{i=1}^n \int _{\gamma _i}\nabla f \cdot d{\varvec{x}}\bigg \vert \\&= \bigg \vert \sum _{i=1}^n \int _{s=x_i}^{s=y_i}\frac{\partial f}{\partial x_i}({\varvec{p}}^{i-1} + (s-x_i){\varvec{e}}_i)ds\bigg \vert \\&\le \sum _{i=1}^n\bigg \vert \int _{s=x_i}^{s=y_i}\frac{\partial f}{\partial x_i}({\varvec{p}}^{i-1} + (s-x_i){\varvec{e}}_i)ds\bigg \vert \\&\le \sum _{i=1}^n \sup _{{\varvec{s}} \in C} \left| \frac{\partial f}{\partial x_i}({\varvec{s}})\right| \vert y_i-x_i\vert \\&\le M\sum _{i=1}^n \vert y_i-x_i\vert \end{aligned}$$

where ${\varvec{e}}_i$ is the ith canonical basis vector. Hence, the required Lipschitz continuity holds with $M = L$. $\square$

### Lemma C.8

#### Lemma C.8

Define the set of functions

$$\begin{aligned} {\mathcal {F}}:= \bigg \{ S_i(t;\epsilon ), I_i(t;\epsilon ),R_i(t;\epsilon ),S^V_i(t;\epsilon ),I^V_i(t;\epsilon ),R^V_i(t;\epsilon ): i \in \{1,...,n\}, \quad \epsilon ,t \ge 0\bigg \}, \end{aligned}$$

where for each fixed $\epsilon$, these functions solve the model equations with parameters

$$\begin{aligned} {\mathcal {P}}= \bigg \{\beta _{ij}^{\alpha }(\epsilon ), \mu _i^{\gamma }(\epsilon ): i,j \in \{1,...,n\}, \quad \alpha \in \{1,2,3,4\}, \quad \gamma \in \{1,2\} \quad \text {and} \quad \epsilon \ge 0\bigg \}, \end{aligned}$$

initial conditions

$$\begin{aligned} {\mathcal {I}}= \bigg \{f(0;\epsilon ): i \in \{1,...,n\}, \quad f \in {\mathcal {F}} \quad \text {and} \quad \epsilon \ge 0\bigg \} \end{aligned}$$

and vaccination policy ${\varvec{U}}(t;\epsilon )$. Suppose that

$$\begin{aligned} \vert p(\epsilon ) - p(0)\vert\le & {} \epsilon \quad \forall p \in {\mathcal {P}}, \\ \vert f_i(0;\epsilon ) - f_i(0;0)\vert\le & {} \epsilon \quad \forall f \in {\mathcal {F}} \end{aligned}$$

and that

$$\begin{aligned} \vert W_i(t,\epsilon ) - W_i(t,0)\vert < \epsilon \quad \forall t \ge 0. \end{aligned}$$

Moreover, suppose that for each $i \in \{1,...,n\}$ and $\epsilon \ge 0$,

$$\begin{aligned} U_i(s;\epsilon ) \ge 0 \quad \text {and} \quad \int _0^t U_i(s;\epsilon ) ds \le N_i \quad \forall t \ge 0. \end{aligned}$$

Then, for each $\delta > 0$ and each $T>0$ there exists some $\eta > 0$ (that may depend on *T* and $\delta$) such that

$$\begin{aligned} \epsilon \in (0,\eta ) \Rightarrow \vert f(t;\epsilon ) - f(t;0)\vert < \delta \quad \forall f \in {\mathcal {F}} \quad \text {and} \quad \forall t \in [0,T] \end{aligned}$$

#### Proof

To begin, it is helpful to note that, by Lemma C.3,

$$\begin{aligned} f(t;\epsilon ) \in [0,\max (N_i)] \quad \forall f \in {\mathcal {F}} \quad \text {and} \quad t \ge 0 \end{aligned}$$

and that, by assumption on the feasibility of $U_i$

$$\begin{aligned} W(t;\epsilon ) \in [0,\max (N_i)] \quad \forall t \ge 0. \end{aligned}$$

Moreover, as the parameter values converge, it can be assumed that

$$\begin{aligned} p(\epsilon ) \in [\alpha ,\beta ] \quad \forall \epsilon \ge 0 \quad \text {and} \quad p \in {\mathcal {P}} \end{aligned}$$

for some $\alpha ,\beta \ge 0$. Moreover, it can be assumed that, as each $\mu _i^{a} > 0$, there is some $\gamma > 0$ such that $\mu _i^{a}(\epsilon ) > \gamma$ for all $\epsilon \ge 0$.

However, there is no condition on the maximal difference (at a point) between $U_i(t;\epsilon )$ and $U_i(t;0)$. To avoid this problem, it is helpful to consider the variable $S^O_i:= S_i + S^V_i$ instead of $S^V_i$. Then, the equations for $S_i$ and $S^O_i$ can be written as

$$\begin{aligned} S_i(t;\epsilon )&= \frac{S_i(0)(N_i - W_i(t;\epsilon ))}{N_i} \exp \left[ -\sum _{j=1}^n\left( \frac{\beta ^1_{ij}(\epsilon )R_j(t;\epsilon )}{\mu ^1_j(\epsilon )} + \frac{\beta ^2_{ij}(\epsilon )R^V_j(t;\epsilon )}{\mu ^2_j(\epsilon )}\right) \right] \\ \frac{dS_i^O(t;\epsilon )}{dt}&= -\sum _{j=1}^n\bigg [ \left( \beta ^1_{ij}(\epsilon )I_j(t;\epsilon )+\beta ^2_{ij}(\epsilon )I^V_j(t;\epsilon )\right) S_i(t;\epsilon )\bigg ] \\&\quad -\sum _{j=1}^n\bigg [\left( \beta ^3_{ij}(\epsilon )I_j(t;\epsilon )+\beta ^4_{ij}(\epsilon )I^V_j(t;\epsilon )\right) (S_i^O(t;\epsilon ) - S_i(t;\epsilon ))\bigg ]. \end{aligned}$$

Then, one can define

$$\begin{aligned} {\varvec{v}}:= ({\varvec{S}}^O,{\varvec{I}},{\varvec{I}}^V,{\varvec{R}},{\varvec{R}}^V)^T \end{aligned}$$

and ${\varvec{p}}(\epsilon )$ to be a vector of the elements of ${\mathcal {P}}$ at some $\epsilon \ge 0$. Then, (substituting for ${\varvec{S}}$), the model equations can be written in the form

$$\begin{aligned} \frac{d {\varvec{v}}(t;\epsilon )}{dt} = \varvec{\Phi }({\varvec{v}}(t;\epsilon ),{\varvec{W}}(t;\epsilon ),{\varvec{p}}(\epsilon )) \end{aligned}$$

where $\Phi$ is a smooth function. Thus, from Lemma C.7, there exists some constant *L* such that, for ${\varvec{v}}$, ${\varvec{W}}$ and ${\varvec{p}}$ within the closed bounded feasible set of values and any $j \in \{1,...,5n\}$,

$$\begin{aligned}{} & {} \vert \varvec{\Phi }({\varvec{v}},{\varvec{W}},{\varvec{p}})_j - \varvec{\Phi }({\varvec{v}}^*,{\varvec{W}}^*,{\varvec{p}}^*)_j\vert \\{} & {} \le L\left( \sum _{i=1}^{5n} \vert v_i-v_i^*\vert + \sum _{i=1}^n\vert W_i - W_i^*\vert + \sum _{i=1}^{4n^2 + 2n}\vert p_i - p_i^*\vert \right) . \end{aligned}$$

Thus, in particular, this means that

$$\begin{aligned} \frac{d}{dt}\bigg (\vert v_j(t;\epsilon ) - v_j(t;0)\vert \bigg )&\le \bigg \vert \frac{d}{dt}\bigg (v_j(t;\epsilon ) - v_j(t;0)\bigg )\bigg \vert \\&\le \bigg \vert \varvec{\Phi }({\varvec{v}}(t;\epsilon ),{\varvec{W}}(t;\epsilon ),{\varvec{p}}(\epsilon ))_i - \varvec{\Phi }({\varvec{v}}(t;0),{\varvec{W}}(t;0),{\varvec{p}}(\epsilon ))_i\bigg \vert \\&\le L\bigg (\sum _{i=1}^{5n} \vert v_i(t;\epsilon )-v_i(t;0)\vert + \sum _{i=1}^n\vert W_i(t;\epsilon ) - W_i(t;0)\vert ...\\&\quad +\sum _{i=1}^{4n^2 + 2n}\vert p_i - p_i^*\vert \bigg ). \end{aligned}$$

Now, adding these 5*n* inequalities together, one seems that

$$\begin{aligned}&\frac{d}{dt}\bigg (\sum _{i=1}^{5n}\vert v_i(t;\epsilon ) - v_i(t;0)\vert \bigg )\\&\quad \le 5nL\left( \sum _{i=1}^{5n} \vert v_i(t;\epsilon )-v_i(t;0)\vert + \sum _{i=1}^n\vert W_i(t;\epsilon ) - W_i(t;0)\vert + \sum _{i=1}^{4n^2 + 2n}\vert p_i - p_i^*\vert \right) \end{aligned}$$

and hence

$$\begin{aligned}&\sum _{i=1}^{5n}\left[ \frac{d}{dt}\left( e^{-5nLt}\vert v_i(t;\epsilon ) - v_i(t;0)\vert \right) \right] \\&\quad \le 5nLe^{-5nLt}\left( \sum _{i=1}^n\vert W_i(t;\epsilon ) - W_i(t;0)\vert + \sum _{i=1}^{4n^2 + 2n}\vert p_i - p_i^*\vert \right) \\&\quad \le (15n^2 + 20n^3)L\epsilon e^{-5nLt}. \end{aligned}$$

Thus, integrating (and using the fact that the initial conditions differ by at most $\epsilon$)

$$\begin{aligned} e^{-5nLt} \sum _{i=1}^{5n}\vert v_i(t;\epsilon ) - v_i(t;0)\vert&\le \sum _{i=1}^{5n}\vert v_i(0;\epsilon ) - v_i(0;0)\vert + (3n+4n^2)\epsilon (1-e^{-5nLt}) \\&\le 5n\epsilon + (3n+4n^2)\epsilon (1-e^{-5nLt}) \end{aligned}$$

which means

$$\begin{aligned} \sum _{i=1}^{5n}\vert v_i(t;\epsilon ) - v_i(t;0)\vert \le 5n\epsilon e^{5nLt} + (3n+4n^2)\epsilon (e^{5nLt} - 1) \end{aligned}$$

and hence, for each $i \in \{1,...,5n\}$

$$\begin{aligned} \vert v_i(t;\epsilon ) - v_i(t;0)\vert \le 5n\epsilon e^{5nLt} +(3n+4n^2)\epsilon (e^{5nLt} - 1). \end{aligned}$$

The right-hand side is non-decreasing in *t* (as $L > 0$) so, taking

$$\begin{aligned} \epsilon < \frac{\delta }{5n e^{5nLt} +(3n+4n^2)(e^{5nLt} - 1)} \end{aligned}$$

ensures that the required inequalities hold for ${\varvec{I}}$, ${\varvec{I}}^V$, ${\varvec{R}}$ and ${\varvec{R}}^V$ for all $s \le t$. Now, note also that $S_i(t;\epsilon )$ is a smooth function of $W_i(t;\epsilon )$, ${\varvec{v}}(\epsilon )$, $S_i(0;\epsilon )$ and ${\varvec{p}}$ so that there exists an $L'$ such that

$$\begin{aligned} \vert S_i(t;\epsilon ) - S_i(0;\epsilon )\vert&< L'\bigg (\sum _{i=1}^{5n} \vert v_i-v_i^*\vert + \sum _{i=1}^n\vert W_i - W_i^*\vert ...\\&\quad + \sum _{i=1}^{4n^2 + 2n}\vert p_i - p_i^*\vert + \vert S_i(0;\epsilon ) - S_i(0;0)\vert \bigg )\\&< L'\epsilon \bigg [5n e^{5nLt} +(3n+4n^2)(e^{5nLt} - 1) + (3n+4n^2) + 1\bigg ]\\&:= \chi (t) \epsilon \end{aligned}$$

and so, as $\chi (t)$ is non-decreasing in *t*, taking

$$\begin{aligned} \epsilon < \frac{\delta }{\chi (t)} \end{aligned}$$

gives the required inequalities for ${\varvec{S}}$ for all times $s \le t$. Finally, note that

$$\begin{aligned} \vert S^V_i(t;\epsilon ) - S^V_i(t;0)\vert&= \vert S^O_i(t;\epsilon ) - S^O_i(t;0) - S_i(t;\epsilon ) + S_i(t;0)\vert \\&\le \vert S^O_i(t;\epsilon ) - S^O_i(t;0)\vert + \vert S_i(t;\epsilon ) - S_i(t;0)\vert \\&\le + 5n\epsilon e^{5nLt} +(3n+4n^2)\epsilon (e^{5nLt} - 1) + \epsilon \chi (t) \end{aligned}$$

and so, as the right-hand side is increasing in *t*, taking

$$\begin{aligned} \epsilon < \frac{\delta }{5n e^{5nLt} +(3n+4n^2)(e^{5nLt} - 1) + \chi (t)} \end{aligned}$$

gives the required inequalities for ${\varvec{S}}^V$ for all times $s \le t$ and hence completes the proof. $\square$
